# Pixel-Level Clustering of Hematoxylin–Eosin-Stained Sections of Mouse and Human Biliary Tract Cancer

**DOI:** 10.3390/biomedicines10123133

**Published:** 2022-12-05

**Authors:** Haruki Inoue, Eriko Aimono, Akiyoshi Kasuga, Haruto Tanaka, Aika Iwasaki, Hideyuki Saya, Yoshimi Arima

**Affiliations:** 1 Hacarus Inc., Kyoto 604-8151, Japan; 2 Clinical and Translational Research Center, Keio University School of Medicine, Tokyo 160-8582, Japan; 3 Department of Cancer Pathology, Faculty of Medicine, Hokkaido University, Sapporo 060-8638, Japan; 4 Department of Hepato-Biliary-Pancreatic Medicine, Cancer Institute Hospital of Japanese Foundation for Cancer Research, Tokyo 135-8550, Japan; 5 Division of Gene Regulation, Institute for Advanced Medical Research, Keio University School of Medicine, Tokyo 160-8582, Japan; 6 Cancer Center, Fujita Health University, Toyoake 470-1192, Japan

**Keywords:** biliary tract cancer, cholangiocarcinoma, pixel clustering, entropy, homology, mouse model

## Abstract

We previously established mouse models of biliary tract cancer (BTC) based on the injection of cells with biliary epithelial stem cell properties derived from KRAS(G12V)-expressing organoids into syngeneic mice. The resulting mouse tumors appeared to recapitulate the pathological features of human BTC. Here we analyzed images of hematoxylin and eosin (H&E) staining for both the mouse tumor tissue and human cholangiocarcinoma tissue by pixel-level clustering with machine learning. A pixel-clustering model that was established via training with mouse images revealed homologies of tissue structure between the mouse and human tumors, suggesting similarities in tumor characteristics independent of animal species. Analysis of the human cholangiocarcinoma tissue samples with the model also revealed that the entropy distribution of cancer regions was higher than that of noncancer regions, with the entropy of pixels thus allowing discrimination between these two types of regions. Histograms of entropy tended to be broader for noncancer regions of late-stage human cholangiocarcinoma. These analyses indicate that our mouse BTC models are appropriate for investigation of BTC carcinogenesis and may support the development of new therapeutic strategies. In addition, our pixel-level clustering model is highly versatile and may contribute to the development of a new BTC diagnostic tool.

## 1. Introduction

Biliary tract cancer (BTC) includes intrahepatic cholangiocarcinoma (IHCC), extrahepatic cholangiocarcinoma (EHCC), and gallbladder carcinoma (GC) [1]. Whereas the incidence of BTC remains highest in parts of Asia and South America, that of IHCC has risen globally over the past two decades [1,2,3]. BTC is often diagnosed at an advanced stage, and patients with advanced BTC have a poor prognosis, with a median survival of ~15 months and a 5-year survival rate of ~2% [1,4,5]. Given that there are few promising anticancer agents for BTC, markers that allow early detection or inform personalized treatment are urgently needed for this malignancy.

Cholangiocarcinoma, also known as bile duct cancer, is a primary malignant epithelial neoplasm that arises from biliary epithelial cells (BECs) lining the bile duct. IHCC and EHCC are thought to develop from two types of intraductal premalignant lesions: biliary intraepithelial neoplasia and intraductal papillary neoplasia of the bile duct [6,7,8]. These two types of carcinomas show similar morphological and immunohistological features. Whether tumors are intra- or extrahepatic, they manifest as well- to moderately differentiated adenocarcinomas, forming glandular or tubular ductlike structures within abundant sclerotic desmoplastic stroma [6,7]. In some instances, other features, such as poorly differentiated-type tissue with pleomorphic cells or signet-ring-type histology are admixed [6,7]. The fibrous stroma shows lymphoplasmacytic infiltration, and areas of purulent inflammation with neutrophilic infiltration are also apparent focally [6,7]. The pathology of BTC is, thus, complex, and pathological diagnosis of tumor tissue requires experience and specialized knowledge based on biological properties.

We have attempted to generate a series of syngeneic mouse models that recapitulate the phenotypes of corresponding human cancer for preclinical studies [9,10]. We previously established BTC (IHCC, GC, and EHCC) mouse models derived from KRAS(G12V)-expressing organoids with biliary epithelial stem cell properties [11]. The organoids were established from EpCAM-positive BECs that were isolated from the intrahepatic bile duct, gallbladder, or extrahepatic bile duct of *Ink4a/Arf*^−/−^ mice. The KRAS(G12V) oncogene was then introduced into the cells of each organoid type, and the resulting cells were injected into syngeneic mice. The KRAS(G12V)-expressing cells formed lethal metastatic adenocarcinomas that appeared to recapitulate the pathological features of human BTC.

The development of whole-slide scanners has made it possible for pathologists to readily generate whole-slide images of histopathology specimens and has prompted the development of techniques for histopathology image analysis [12]. An increasing number of studies have applied deep-learning techniques to perform segmentation and classification of histopathology images [13,14]. However, many deep-learning approaches require a set of training images and labels or detailed annotations for classification and semantic segmentation. In supervised learning models, labels and annotations are generated by pathologists, but, in general, it is difficult to perform the large amount of labeling and annotation required to train a deep-learning model. On the other hand, unsupervised learning methods avoid the use of annotations, with one such method being clustering analysis. Clustering, with or without deep-learning techniques, is used for feature grouping. Given the black-box nature of deep learning and the processing speed required for large images, the KMeans clustering method—a type of unsupervised learning adopted for unlabeled data—is potentially useful for histopathologic analysis.

In the present study, we have analyzed hematoxylin–eosin (H&E)-stained images of mouse-BTC-model tumor tissue, as well as of human cholangiocarcinoma specimens by pixel-level clustering with the use of KMeans. Our cluster analysis revealed homologies of tissue structure between the mouse tumors and human cholangiocarcinoma, providing further support for the suitability of our mouse BTC models for preclinical studies. In addition, the entropy of pixels allowed us to distinguish between cancer and noncancer tissue areas in histopathological specimens. Our clustering model, therefore, has the potential to support future cancer research based on histopathologic specimens.

## 2. Materials and Methods

### 2.1. Mouse BTC Tissue Images

The BTC mouse model was established as previously described [11]. At 4 weeks after intrahepatic injection of KRAS(G12V)-expressing *Ink4a/Arf*^−/−^ mouse intrahepatic-bile-duct (IHBD)-derived BECs (5 × 10^4^ cells) in wild-type (WT) C57BL/6J mice, tumor tissue was removed, fixed with 4% paraformaldehyde, embedded in paraffin, and sectioned, and the sections were then depleted of paraffin and stained with H&E. The tissue sections were observed with a BZ-X810 microscope (Keyence, Osaka, Japan) and were subjected to digital image scanning with NDRscan3.2.4 software (Hamamatsu Photonics, Hamamatsu, Japan). The H&E images were acquired at a resolution of 0.441 μm/pixel and saved as ndpi files.

### 2.2. Human Cholangiocarcinoma Tissue Images

A Cholangiocarcinoma Tissue Microarray (69572620) was obtained from TriStar Technology Group (Washington, DC, USA). The sections were depleted of paraffin, stained with H&E, observed with a BZ-X810 microscope (Keyence), and subjected to a digital image scan with NDRscan3.2.4 software (Hamamatsu Photonics). The H&E images were acquired at a resolution of 0.441 μm/pixel and saved as ndpi files.

### 2.3. Clustering-Based Analysis of H&E Images

Multiscale basic features (MBFs), which can represent the texture of an image, were used as features for clustering. The parameters adopted as MBFs are shown in Table 1. The KMeans model with a parameter of 30 clusters was used as the clustering model. All procedures were performed with the Python packages scikit-image and scikit-learn [15,16].

### 2.4. Entropy

**Figure 1:**
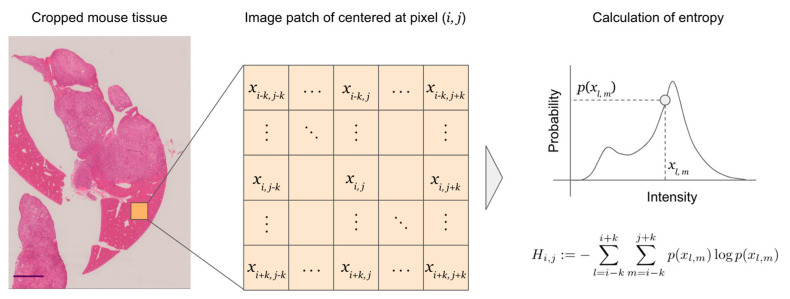
Example of local entropy calculation at pixel (*i, j*) of an image patch centered at pixel (*i, j*). The same calculation is performed on all pixels in the tissue. *k* was set to 20. Scale bar = 2.0 mm.

Entropy was calculated for each pixel as follows [17,18]:

$$H_{i,\;j}\;:=-\overset{i+k}{\underset{l=i-k}{\sum }}\overset{j+k}{\underset{m=j-k}{\sum }}p\left(x_{l,\;m}\right)\log p\left(x_{l,\;m}\right)$$

where *H_i, j_* is the local entropy of coordinates (*i, j*) in the image, *x_l, m_* is the signal intensity at coordinates (*l, m*), *p*(⋅) is the probability, and *k* is a constant that represents the range of surrounding pixels considered in the calculation of entropy at coordinates (*i, j*) (Figure 1).

## 3. Results

### 3.1. Image Comparison for Mouse BTC Tumor and Human Cholangiocarcinoma Tissue

We established KRAS(G12V)-expressing BTC-initiating cells from BECs derived from the IHBD of *Ink4a/Arf*^−/−^ mice [11]. These cells formed tumors after intrahepatic injection into syngeneic WT mice (Supplementary Figure S1). The gross appearance of the liver and H&E staining of liver sections from injected mice revealed the presence of multiple nodules consisting of moderately differentiated adenocarcinoma with cuboidal cells and a ductular component without mucin production. Biliary intraepithelial neoplasia was apparent in the dilated IHBD. A poorly differentiated adenocarcinoma component was also evident, scattered within the nodules. The tumors had a sclerotic fibrous stroma with inflammatory cells. In addition, small abscesses with eosinophilic infiltration were scattered throughout the nodules, and highly lymphoplasmacytic infiltration was evident at interface regions. The adenocarcinoma and fibrous stroma of the mouse BTC model thus mimic the characteristics of human cholangioadenocarcinoma (Supplementary Figure S1), although the mouse tumors show a relatively poor demarcation because of the mild indolence of background hepatocytes compared with human hepatocytes.

**Figure 2:**
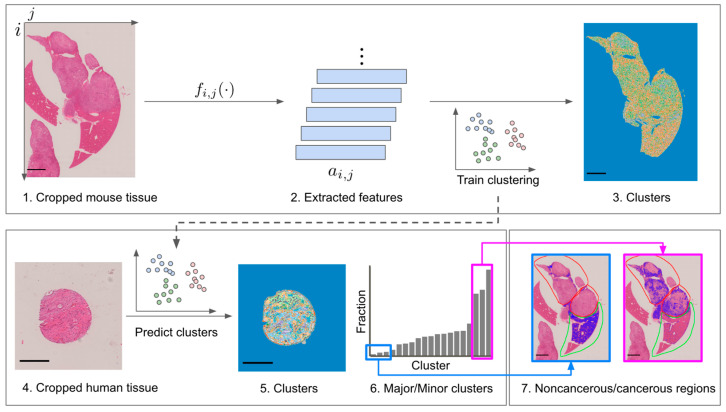
Overview of homology analysis for mouse and human histopathologic images (*n* = 5 and *n* = 90, respectively). Multiscale basic features (MBFs) were extracted for each pixel in cropped images of mouse tissue (see Section 2), after which KMeans clustering was performed with a cluster size of 30. Major/Minor clusters were obtained from predicted clusters for human tissue. Scale bars are 2.0 mm for mouse tissue images and 0.5 mm for human tissue images.

Given that mice and humans are both mammals but different species, a direct comparison of the two reveals species differences, rather than essential homologies or differences. To overcome this problem, we developed an indirect homology analysis method based on clustering to reveal homologies between mouse and human histopathologic images (Figure 2). In the subsequent subsections, we describe the clustering of human images by a clustering model trained with mouse images, as well as the relation between clusters and clinical information.

### 3.2. Pixel-Level Clustering

Clustering-based homology is defined in the present study as morphological similarity between regions of the same cluster ID in different images. For example, cluster 4 indicates a cancerous region in both mice and humans. In the present study, a clustering model trained with mouse images was applied to human images in order to evaluate homology in a qualitative manner.

**Figure 3:**
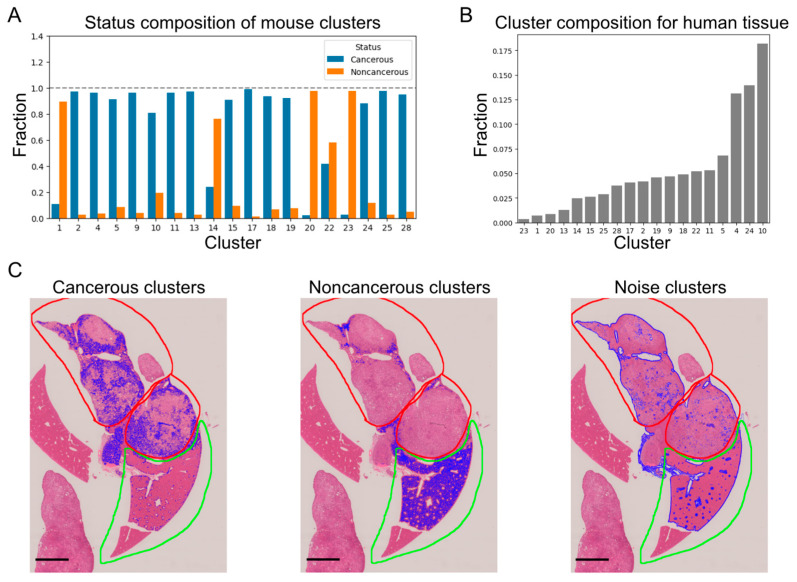
Pixel-level clustering. (**A**) Cancerous/noncancerous composition for clusters in mouse tissue. (**B**) Cluster composition for human tissue microarray specimens. (**C**) Clusters and annotations of mouse tissue. Areas colored blue indicate cancerous, noncancerous, or noise-cluster regions. Red and green contours indicate annotation of cancerous and noncancerous regions, respectively, by a medical expert. Scale bar = 2.0 mm.

A clustering model with a cluster size of 30 was trained with mouse images, and the model was then applied to each of the mouse and human images to calculate the cluster composition ratio (Figure 3A,B). Noise clusters, such as clusters consisting only of tissue edges and including clusters 3, 6, 7, 8, 12, 16, 21, 26, 27, 29, and 30, were visually excluded (Figure 3C). 

In mice, most clusters consisted largely of cancerous regions (Figure 3A), with only clusters 1, 14, 20, and 23 being composed largely of noncancerous regions. Most of the regions in the human tissue specimens on the microarray were cancerous regions. Clusters with a relatively large composition ratio in the human images were, therefore, considered to represent human cancerous clusters, whereas those with a relatively small composition ratio were considered to represent noncancerous regions (Figure 3B). We extracted the clusters with the top three and bottom three cluster composition ratios for the human images (Figure 3B) and compared them with the clusters with the same cluster IDs in the mouse images (Figure 3C, Supplementary Figures S2 and S3). The top three clusters for the mouse images were clusters 4, 10, and 24, respectively, and are representative clusters of cancerous regions in mice (Figure 3C, Supplementary Figure S2). Similarly, the bottom three clusters for the mouse images were 1, 20, and 23 and are representative clusters of noncancerous regions in mice (Figure 3C, Supplementary Figure S2). The fact that the clustering model trained with mouse images was able to capture representative cancer and noncancerous structures in human histopathology images in a qualitative manner was indicative of a certain level of homology between mouse and human histopathology images.

### 3.3. Relation between Image Features and Clinical Information

We next examined the relation between the extracted clusters and clinical information accompanying the human tissue microarray. Given that the clustering model was trained with mouse images, this analysis allowed an indirect and quantitative evaluation of the relation between morphological features of mouse histopathology images and human clinical information. We focused on entropy [17,18], a feature of data clutter that can be interpreted as the disorderly nature of cancer, as an image feature that can be quantified at the pixel level and is interpretable.

**Figure 4:**
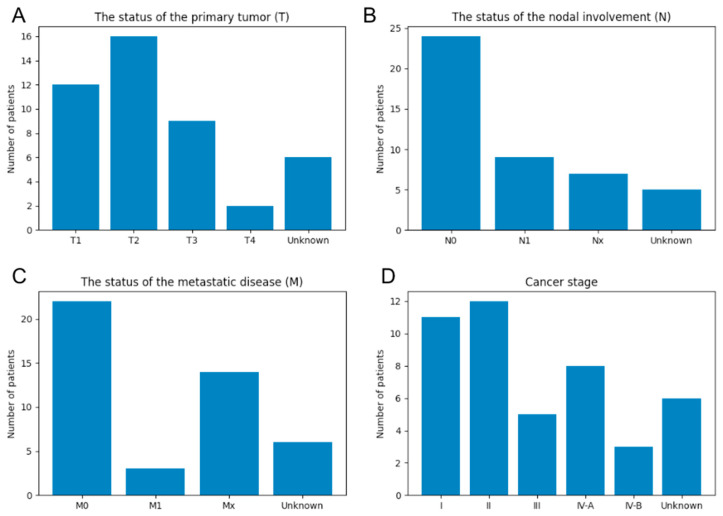
TNM classification (**A**–**C**) and cancer stage derived from the 7th edition of the UICC classification (**D**) for human tissue microarray specimens.

Given that the provided clinical information included TNM classification (Figure 4A–C), we generated cancer-stage information with the use of the 7th edition of the UICC (Union for International Cancer Control) classification (Figure 4D). Data with no or an unknown TNM label were assigned to an “Unknown” classification and were excluded from subsequent analysis.

In Section 3.3.1, we compare the entropy of noncancerous regions (clusters 1, 20, and 23) and cancerous regions (clusters 4, 10, and 24) for mouse and human, whereas we compare the entropy of noncancerous and cancerous regions for human tumors at each stage in Section 3.3.2.

#### 3.3.1. Entropy Comparison of Cancer and Noncancer Regions in Mice and Humans

**Figure 5:**
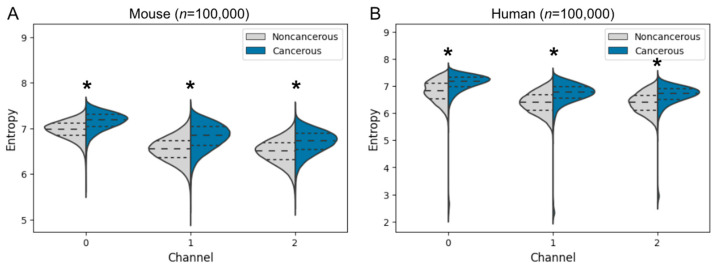
Entropy of pixels in each channel for noncancerous and cancerous regions of mouse (**A**) and human (**B**) histopathology images. Channels 0, 1, and 2 correspond to red, green, and blue, respectively. Dashed lines indicate the median, and dotted lines the first and third quartiles. * *p* < 0.001 (Welch’s *t*-test).

Given that the area of tissue slices differed among histopathology images, pixel-wise entropy was resampled equally from each noncancerous and cancerous region of each tissue specimen with the use of the inverse of tissue area as a resampling weight (*n* = 100,000). The entropy distributions were significantly higher for cancerous regions than for noncancerous regions in all color channels for both mice and humans, and they were higher in the red channel than in the other channels (Figure 5). Thus, entropy comparison may be useful in distinguishing cancer from noncancer regions. The tumor microenvironment (TME) surrounding tumor cells comprises immune cells, fibroblasts, endothelial cells, and a wide range of soluble factors. The TME of cholangiocarcinoma is characterized by an abundant desmoplastic stroma and shows a high heterogeneity [19,20]. Pixel-wise entropy may capture tumor heterogeneity, which plays a central role in cancer progression and resistance to anticancer therapies [19,20,21]. Furthermore, the fact that the clustering model was trained with mouse histopathology images and yet a similar trend was also obtained for humans with respect to pixel-wise entropy suggests that there is a morphological similarity between mouse and human histopathology images.

#### 3.3.2. Entropy Comparison of Cancer and Noncancer Regions at Each Cancer Stage in Human Specimens

**Figure 6:**
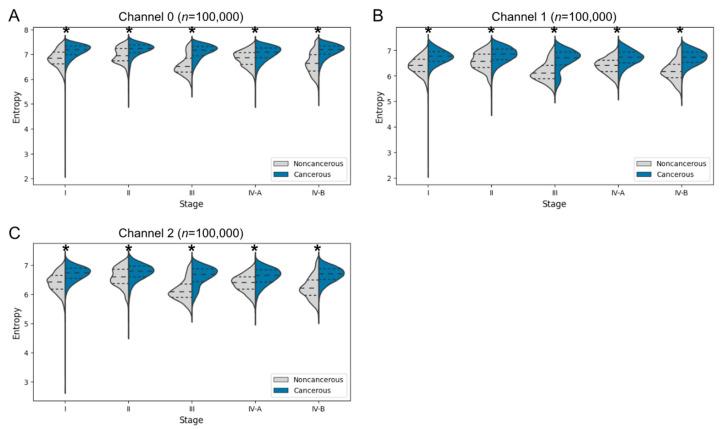
Pixel-wise entropy in red (**A**), green (**B**), and blue (**C**) channels at each cancer stage for cancerous and noncancerous regions of human specimens. Dashed lines indicate the median, and dotted lines the first and third quartiles. * *p* < 0.001 (Welch’s *t*-test).

As in the previous analysis, pixel-wise entropy was resampled equally for human tumors at each stage with the use of the inverse of tissue area as a weight (*n* = 100,000). The extracted entropy distributions were grouped by cancer stage and compared between noncancerous and cancerous regions (Figure 6). The entropy distributions were significantly higher for cancerous regions than for noncancerous regions in all color channels, supporting the notion that entropy is a potentially useful indicator to distinguish cancer from noncancer areas. Of note, the distribution for noncancerous regions was biased toward the low-entropy side for stages III and IV-B. Histograms of entropy showed that the distribution tended to change between stages to a greater extent in the noncancer regions than in the cancer regions, suggesting that the high-entropy portions of noncancerous regions became incorporated into the entropy distribution of cancerous regions as a result of cancerous transformation.

**Figure 7:**
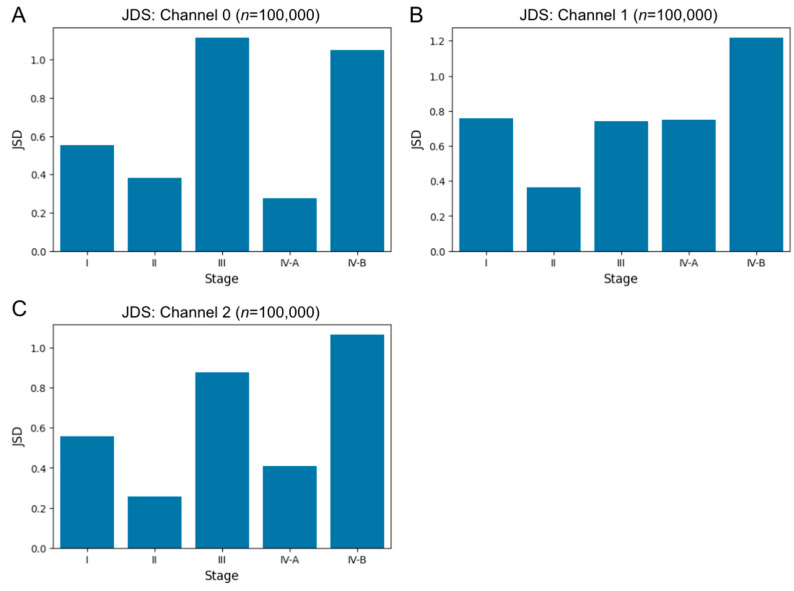
Jensen–Shannon divergence (JSD) of pixel-wise entropy in red (**A**), green (**B**), and blue (**C**) channels between cancerous and noncancerous regions at each cancer stage for human specimens.

We also calculated the Jensen–Shannon divergence (JSD) between the entropy distributions of the noncancerous and cancerous regions for each stage (Figure 7). The JSD is a statistical method of divergence measures based on the Kullback–Leibler divergence [22,23], and JSD values indicate a difference in distribution; a value near zero indicates similarity between two probability distributions, whereas higher values of JSD indicate a greater divergence. The JSD values between the entropy distributions of the noncancerous and cancerous regions did not show the similarity. Although JSD did not show a monotonic change with cancer stage progression, JSD values of stage IV-B were larger than that of stage I in all color channels.

## 4. Discussion

Here we analyzed H&E images of mouse BTC and human cholangiocarcinoma tissue using pixel-level clustering. We had previously generated a series of syngeneic mouse models for various types of cancer that recapitulate the phenotypes of the corresponding human malignancies [9], including several derived from epithelial cells, such as lung adenocarcinoma [10] and BTC [11]. With a clustering model trained with mouse tissue images, we have now compared inference results between mouse and human images and found that the model is capable of distinguishing cancerous from noncancerous regions.

We quantified pixel-wise entropy and found that the entropy distribution was significantly higher in cancerous regions than in noncancerous regions of both mouse and human tumor specimens, suggesting that entropy is a useful indicator to distinguish cancerous from noncancerous regions. The characteristics of cancer tissue have previously been determined visually by experienced pathologists. Diagnostic procedures will become more efficient when techniques for histopathology image analysis have developed to a stage that allows for the automated extraction of cancer features from such images. Furthermore, a system that allows for the automatic detection of cancer cell clusters in surgically removed tissue will be highly beneficial for genomic analysis. However, tumors are heterogeneous and complex tissues. The TME of cholangiocarcinoma comprises abundant fibroblasts in the desmoplastic stroma, as well as immune cells, endothelial cells, and various other factors. It also manifests a high level of heterogeneity that plays a central role in cancer progression and resistance to anticancer therapies [19,20]. Our pixel-wise entropy analysis may capture this tumor heterogeneity. Such heterogeneity is due not only to the TME but also to the diversity of cancer cells at genetic and epigenetic levels [19,20,21], and it is expected to be amenable to spatiotemporal analysis through combined evaluation of pathological images and gene and molecular profiles [24].

BTC has been treated with cytotoxic agents, such as gemcitabine, cisplatin, and S-1, and a recent phase III study found an overall survival benefit for upfront treatment with immunotherapy plus chemotherapy in patients with advanced BTC [25]. Given that there are few promising anticancer agents for BTC, however, new therapeutic options are needed. Even if potential drugs prove to be effective for treatment of cancer cells transplanted into mice, they are not guaranteed to also be effective in humans. Exploration of the homology between human and mouse cancers may therefore lead to an improved efficiency of drug discovery. With the use of an unsupervised pixel-level-clustering method, we have now shown that one of our mouse BTC models recapitulates human cholangiocarcinoma, indicating that these models are suitable for further studies of human BTC and should facilitate drug discovery, including that of TME-targeted agents.

In addition to the comparison of mouse and human tumor tissues, we analyzed entropy distribution according to cancer stage for the human tissue microarray specimens and revealed variability in such a distribution among stages for noncancerous regions. Of interest, the noncancerous regions of late-stage human cholangiocarcinoma tended to have a broader range of entropy values. Recent studies have revealed the clonal expansion of noncancerous cells with multiple gene mutations in regions adjacent to cancer tissue [26], with these cells possibly contributing to changes in entropy values. In particular, the distribution was biased toward the low-entropy side for stages III and IV-B, suggesting that the high-entropy portions of noncancerous regions undergo transformation to cancer. The lack of a similar variation in entropy distribution for cancerous regions may be due to the larger area of these regions relative to noncancerous regions for the human specimens. Further studies with specimens consisting of equal areas of noncancerous and cancerous regions are warranted.

For a pixel-level-clustering method, we adopted KMeans clustering, a fast and scalable method that does not require expert annotation. With the use of MBFs [15], which are linear features amendable to analysis by many algorithms, we showed that KMeans clustering was able to distinguish cancerous from noncancerous regions in histopathology images, suggesting that our model can capture pathological features. On the other hand, KMeans is not suitable for clustering of nonlinear features. Given that histopathology images are complex, it would be desirable to introduce nonlinearity into the model, which has been achieved in recent studies with the use of deep learning [27,28,29,30]. Such approaches train models that transform nonlinear features into linearly separable features and cluster them using fast algorithms, such as KMeans. Clustering methods also generally have problems with interpretability of semantic concepts for each cluster. A recent study used a self-organizing map (SOM) to cluster multiplexed stained tissue images [31]. An SOM is a dimensionality-reduction algorithm that can map and group high-dimensional features into a low-dimensional space, but, like clustering, it learns similarities among data and does not learn semantic concepts. Another recent study revealed a relation between intratumoral heterogeneity and patient survival in non-small-cell lung cancer by pixel-level clustering [32]. The results showed that morphological features derived from images alone contained pathological information, but experts were necessary for interpretation of the patterns. An effective approach to this problem is unsupervised semantic segmentation.

Unsupervised semantic segmentation not only performs pixel-level clustering, but also assigns a semantic concept to each cluster. The self-supervised Vision Transformer (ViT) was shown to be useful for unsupervised semantic segmentation [33], and research has recently been conducted to incorporate ViT into unsupervised semantic segmentation tasks [34]. The use of such techniques should allow pathologically important features to be obtained through unsupervised learning. Given the absence of human intervention, novel pathological features might be found that have not been noticed by humans, which may be highly beneficial for diagnosis.

## 5. Conclusions

We analyzed H&E images of mouse BTC model tumor tissue and human cholangiocarcinoma tissue by pixel-level clustering with machine learning. Our cluster analysis revealed homologies of tissue structure between the mouse and human tumors, indicating that our mouse BTC models are appropriate for preclinical studies. The entropy of pixels allowed us to distinguish between cancer and noncancer areas of tissue specimens. Our clustering-based tissue analysis may provide the basis for development of a new diagnostic tool and new therapeutic strategies for BTC.

## Figures and Tables

**Table 1:** Parameters used as MBFs.

Parameter	Value
Intensity	True
Edges	False
Sigma_min	3
Sigma_max	7
Multichannel	True

## Data Availability

The data presented in this study are available upon request from the corresponding author. All code for data processing and analysis associated with the current submission is available at https://github.com/hacarus/pixel-level-clustering-btc (accessed on 1 December 2022).

## Supplementary Materials

The following supporting information can be downloaded at https://www.mdpi.com/article/10.3390/biomedicines10123133/s1. Supplementary Figure S1: H&E staining images of tumor tissue. Supplementary Figure S2: Clusters and annotations of mouse tissue. Supplementary Figure S3: Clusters and annotations of human cholangiocarcinoma tissue specimens.
