# Supplementary material for: Pixel-Level Clustering of Hematoxylin–Eosin-Stained Sections of Mouse and Human Biliary Tract Cancer
Source: Biomedicines. 2022 Dec 5;10(12):3133. doi: 10.3390/biomedicines10123133 (PMC9775647; doi:10.3390/biomedicines10123133)
Supplement: Supplementary file 1 [file biomedicines-10-03133-s001.zip › biomedicines-1965824-supplementary.pdf]

A B

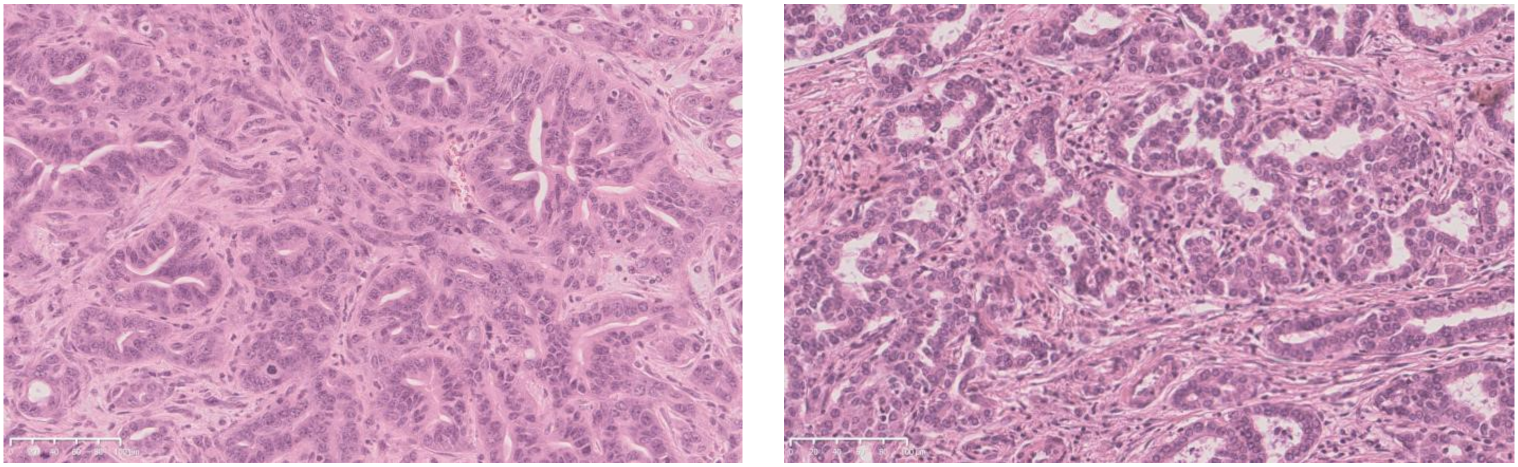

Figure S1. H&E staining images of tumor tissue. (**A**)Tumor formed 4 weeks after intrahepatic injection of KRAS(G12V)-expressing *Ink4a/Arf*$^{-/-}$ mouse IHBD-derived BECs (5 × 10^4 cells) in WT syngeneic mice. Scale bar, 100 μm. (**B**) Tumor of a patient with intrahepatic cholangiocarcinoma from the tissue microarray (22,13D44885). Scale bar, 100 μm.

A

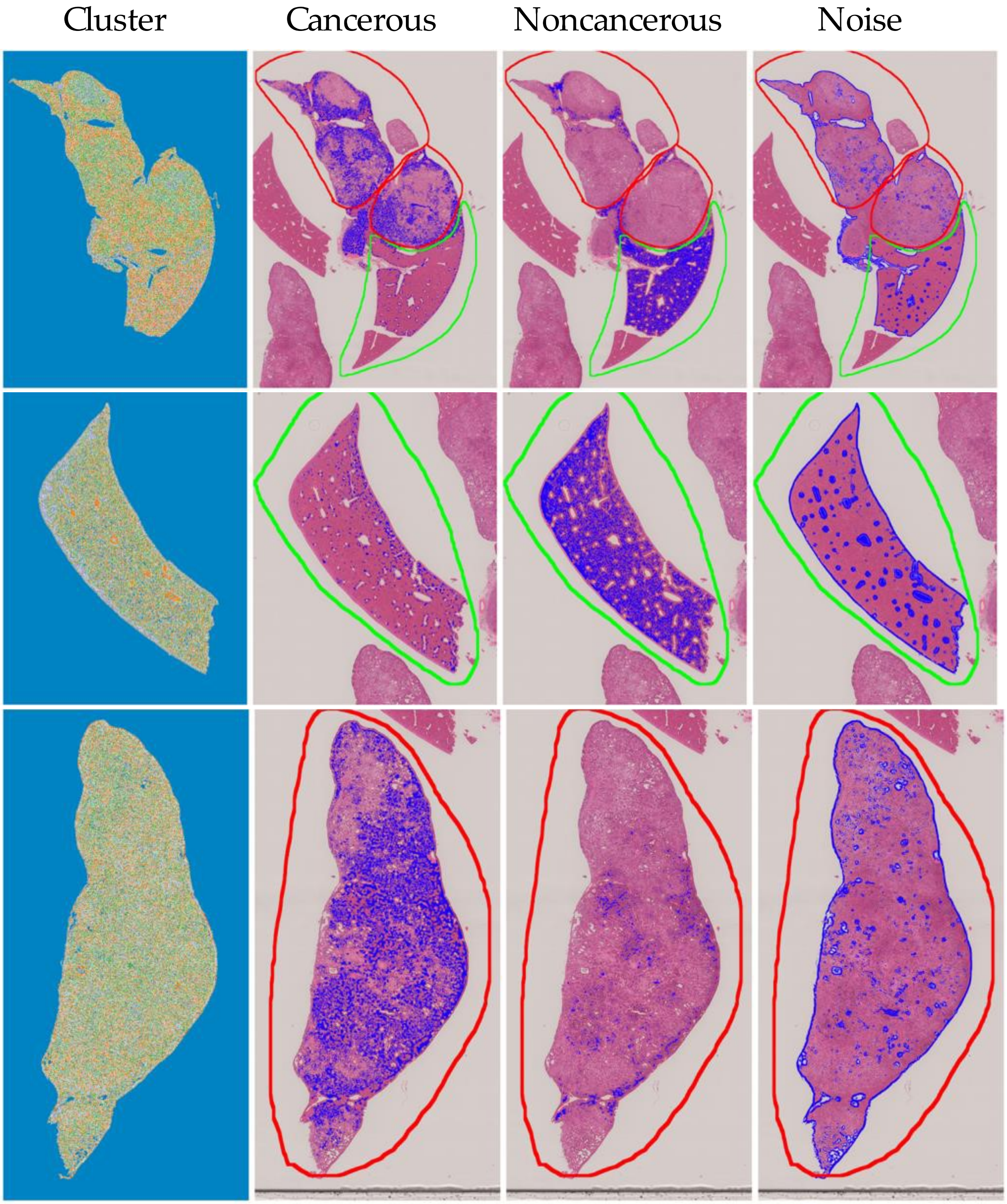

Figure S2. Clusters and annotations of mouse tissue. In the Cluster column, different colors indicate different clusters. In the Cancerous, Noncancerous, and Noise columns, the areas filled with blue indicate the corresponding clusters. Red and green contours indicate annotations of cancerous and noncancerous regions, respectively. Each annotation was made by a medical expert.

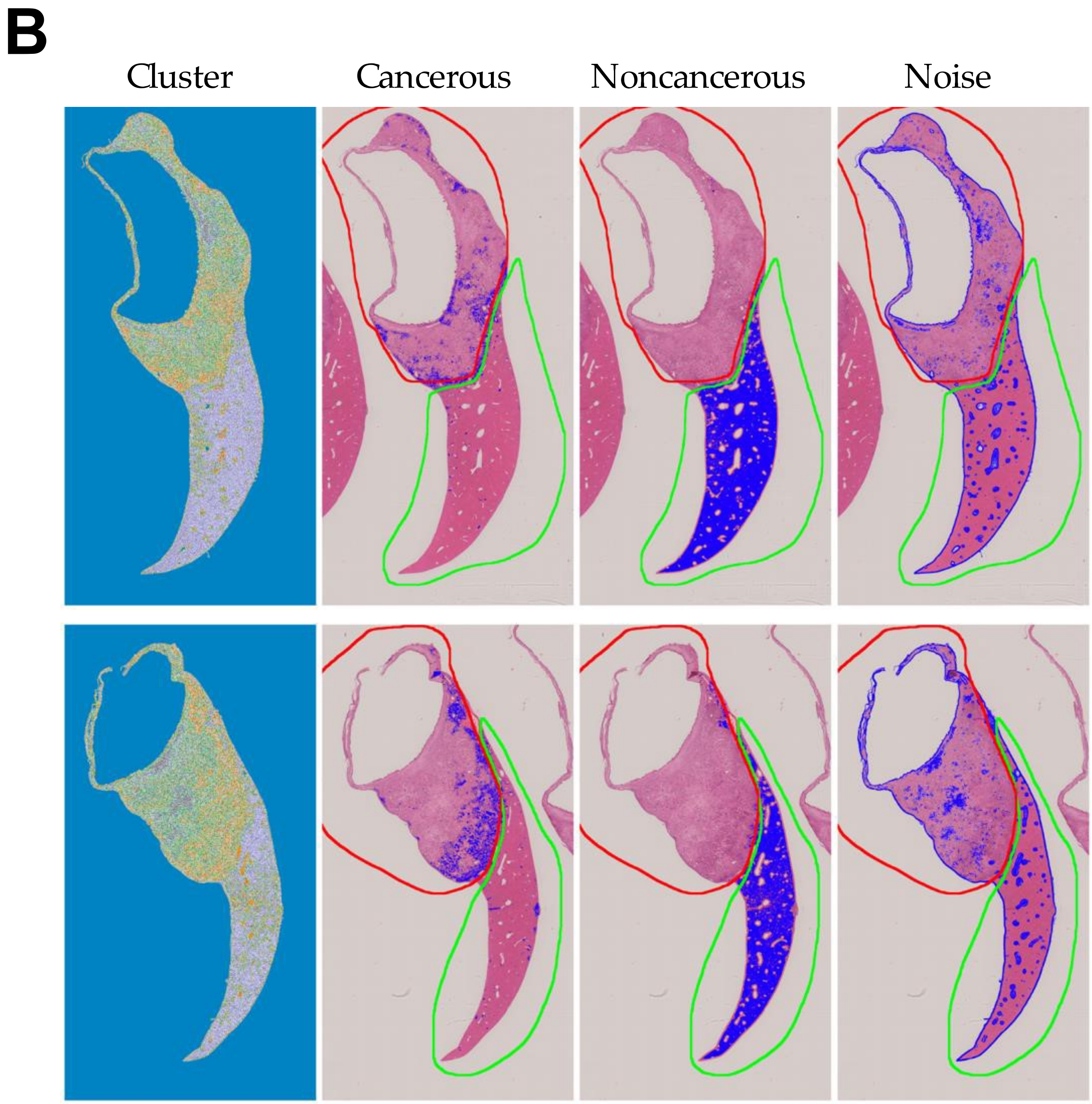

Figure S2. Clusters and annotations of mouse tissue. In the Cluster column, different colors indicate different clusters. In the Cancerous, Noncancerous, and Noise columns, the areas filled with blue indicate the corresponding clusters. Red and green contours indicate annotations of cancerous and noncancerous regions, respectively. Each annotation was made by a medical expert.

A

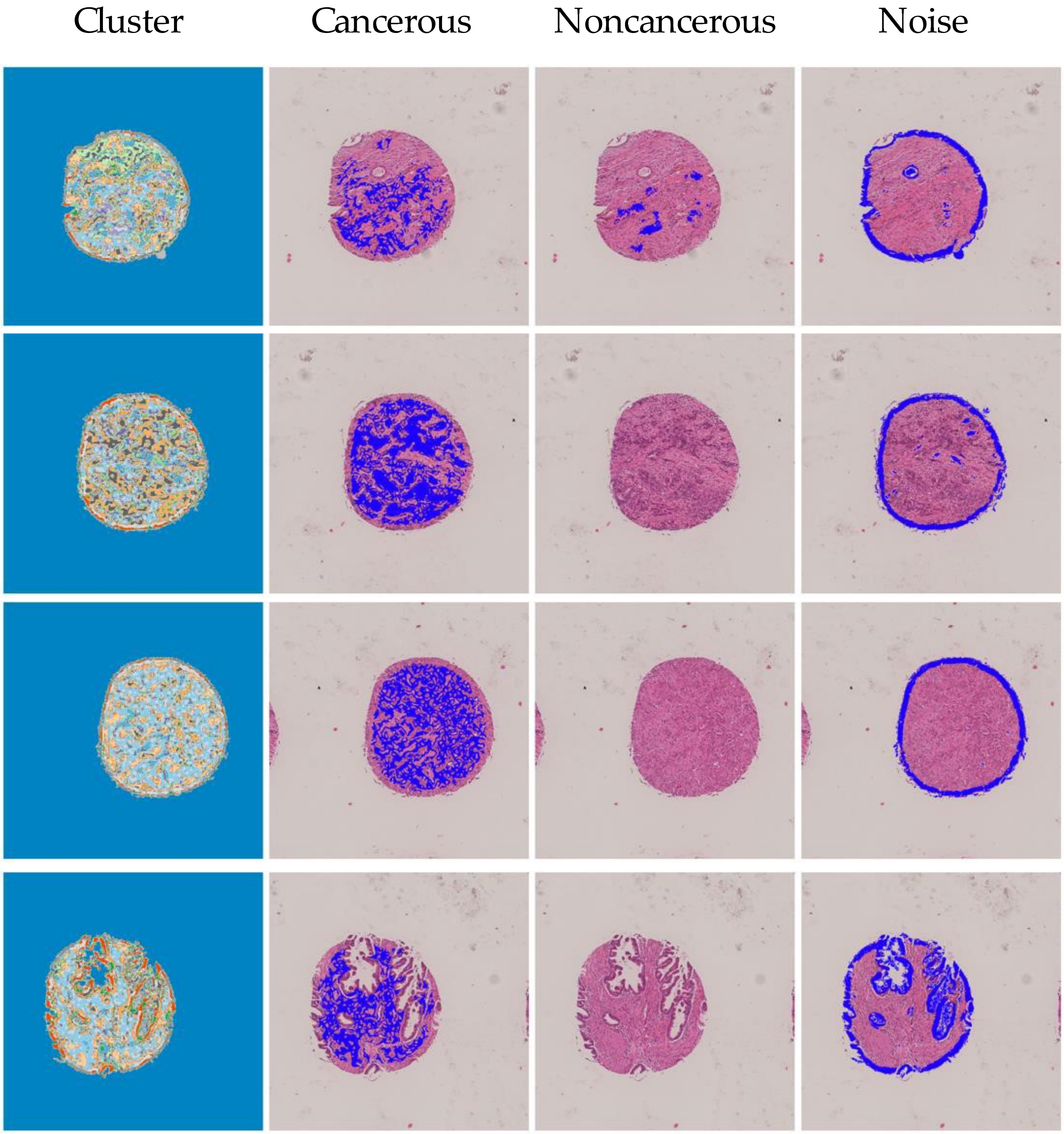

Figure S3. Clusters and annotations of human cholangiocarcinoma tissue specimens. In the Cluster column, different colors indicate different clusters. In the Cancerous, Noncancerous, and Noise columns, the areas filled with blue indicate the corresponding clusters. Red and green contours indicate annotations of cancerous and noncancerous regions, respectively. Each annotation was made by a medical expert.

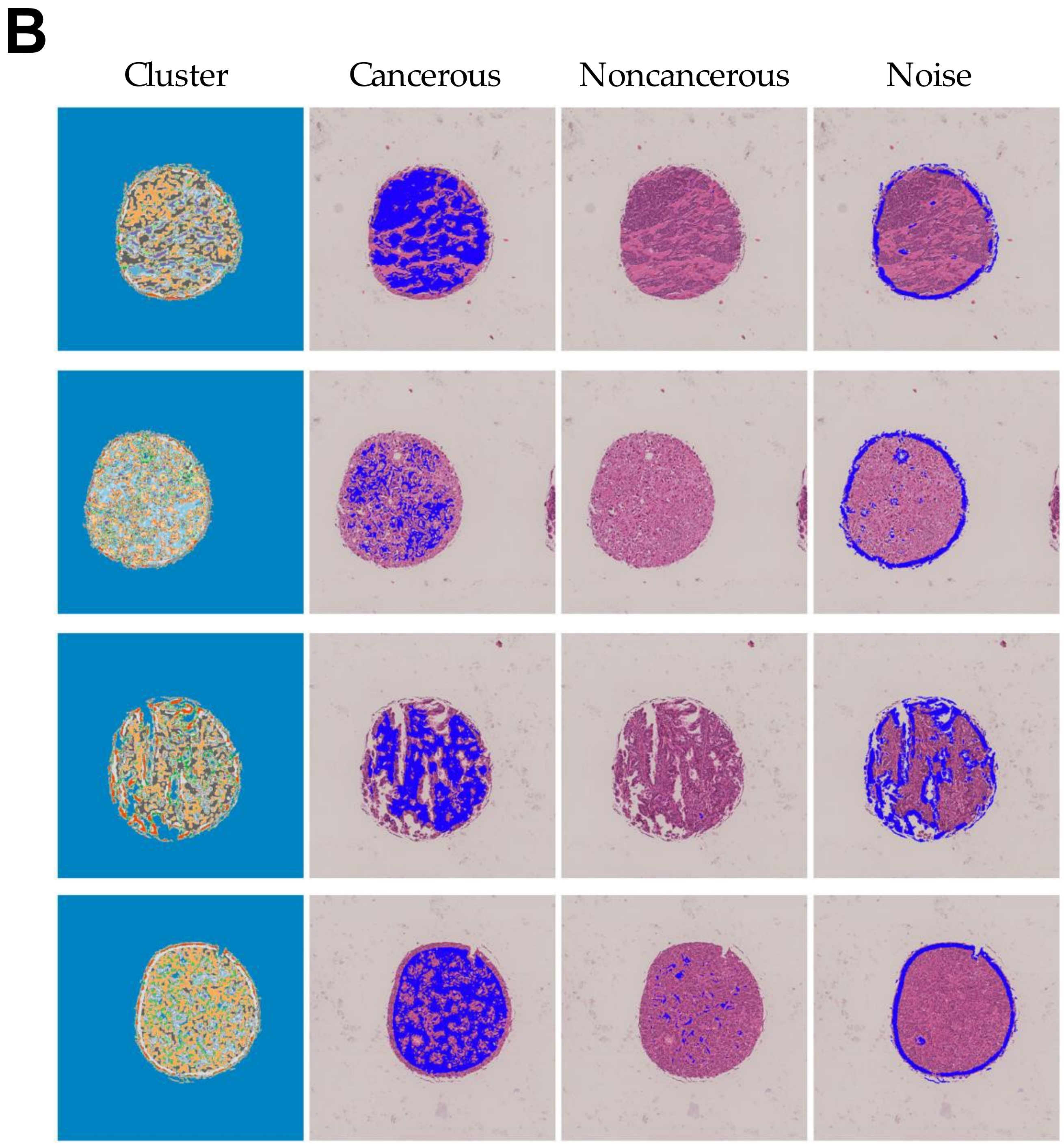

Figure S3. Clusters and annotations of human cholangiocarcinoma tissue specimens. In the Cluster column, different colors indicate different clusters. In the Cancerous, Noncancerous, and Noise columns, the areas filled with blue indicate the corresponding clusters. Red and green contours indicate annotations of cancerous and noncancerous regions, respectively. Each annotation was made by a medical expert.

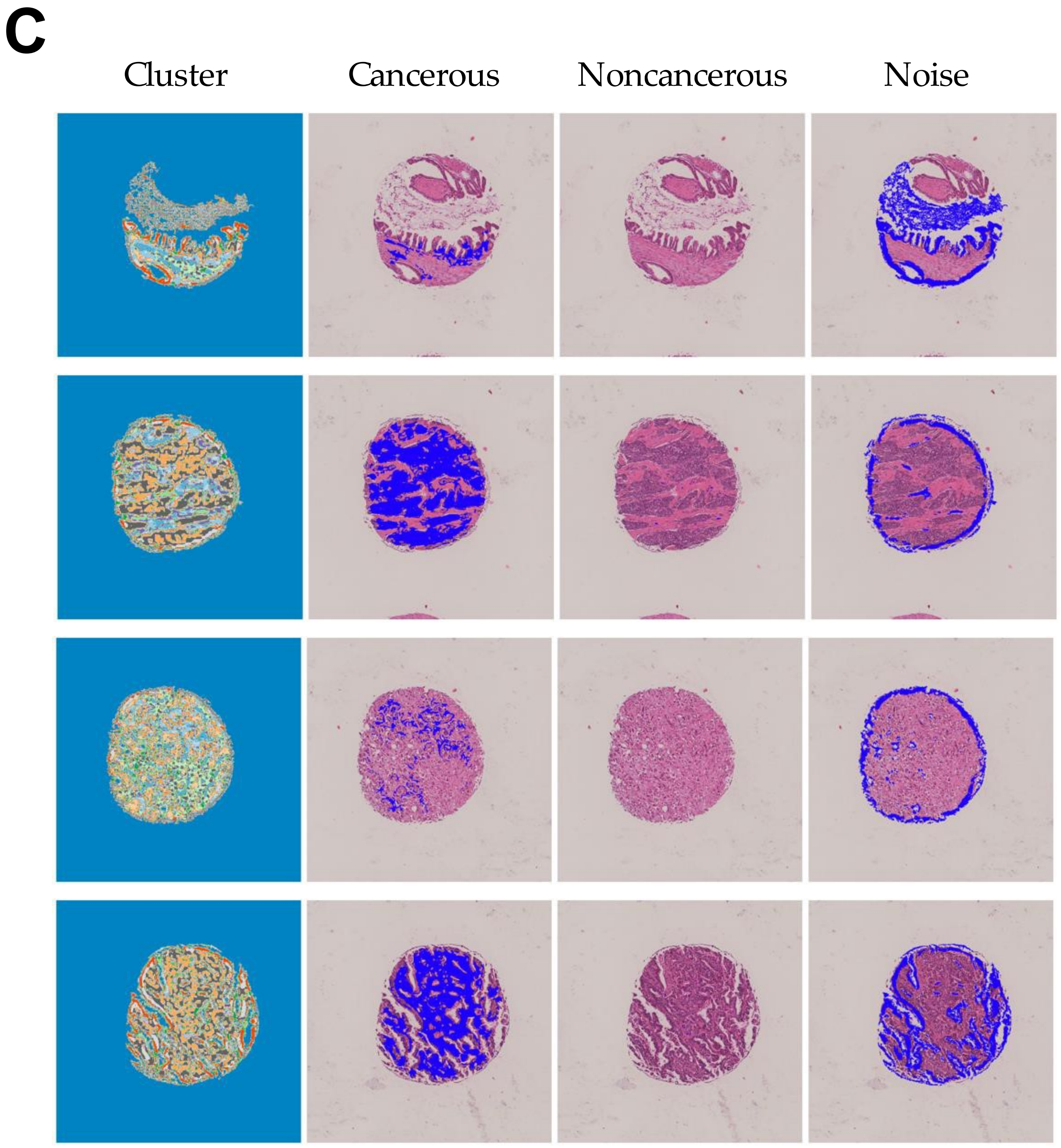

Figure S3. Clusters and annotations of human cholangiocarcinoma tissue specimens. In the Cluster column, different colors indicate different clusters. In the Cancerous, Noncancerous, and Noise columns, the areas filled with blue indicate the corresponding clusters. Red and green contours indicate annotations of cancerous and noncancerous regions, respectively. Each annotation was made by a medical expert.

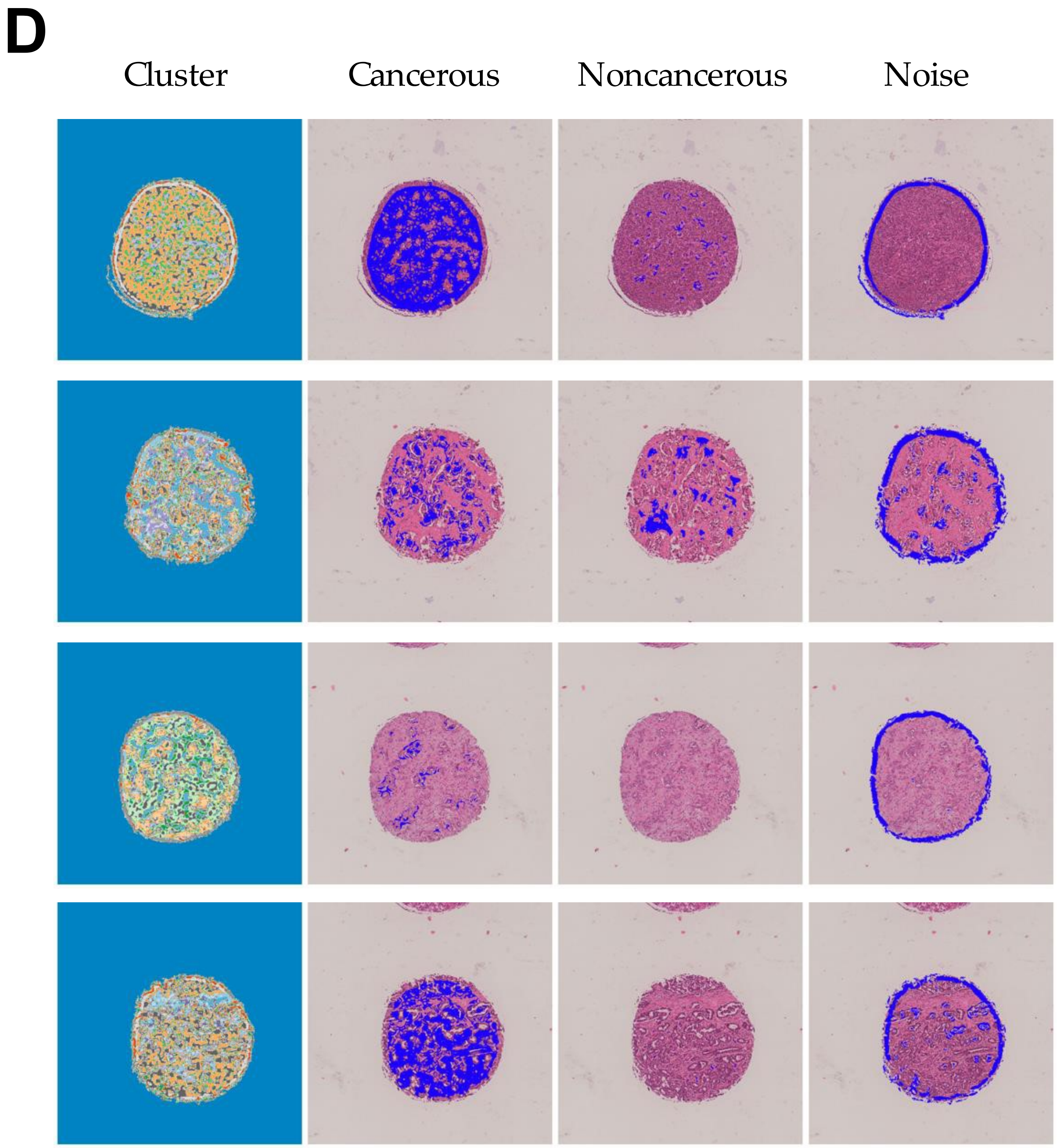

Figure S3. Clusters and annotations of human cholangiocarcinoma tissue specimens. In the Cluster column, different colors indicate different clusters. In the Cancerous, Noncancerous, and Noise columns, the areas filled with blue indicate the corresponding clusters. Red and green contours indicate annotations of cancerous and noncancerous regions, respectively. Each annotation was made by a medical expert.

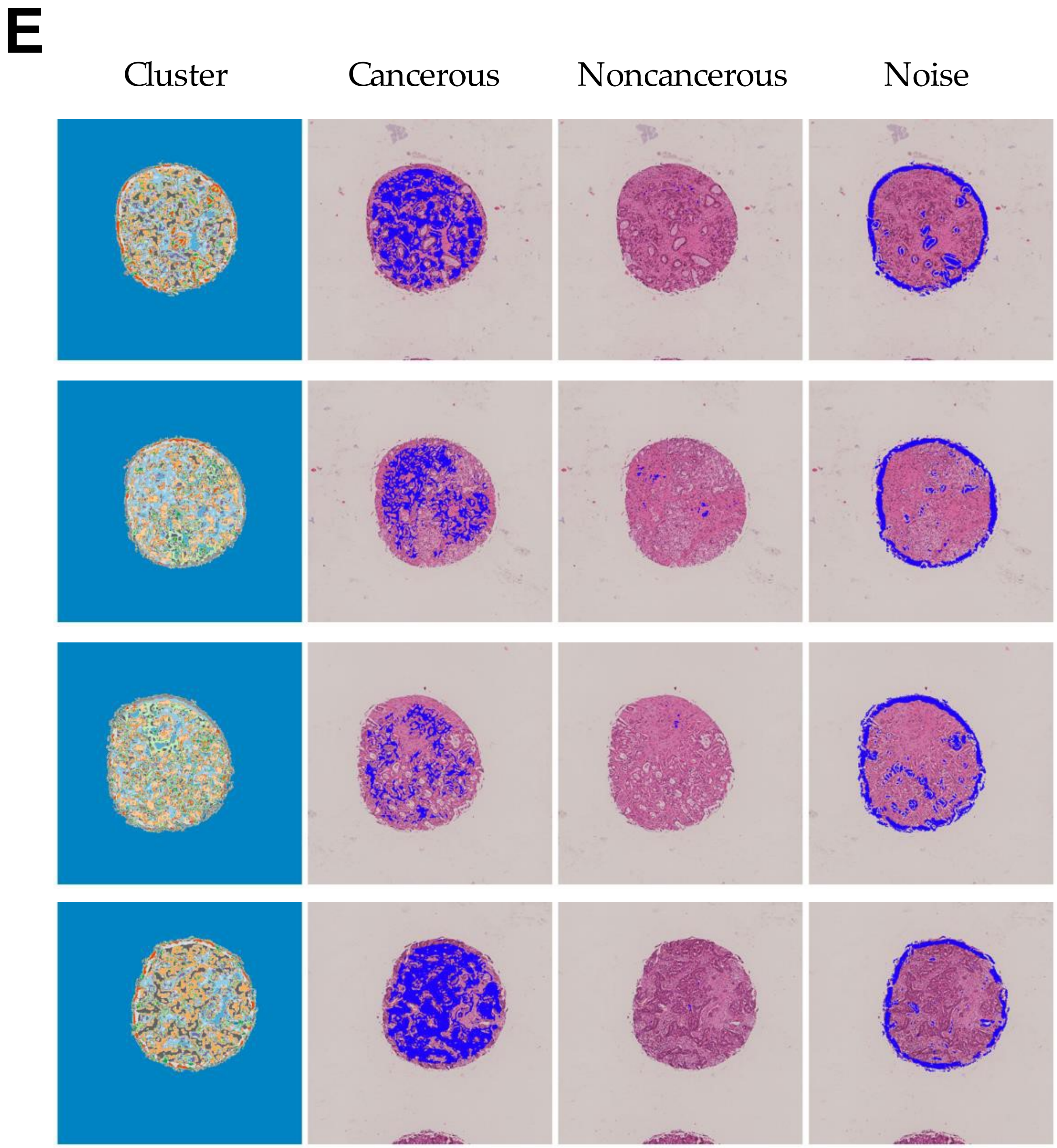

Figure S3. Clusters and annotations of human cholangiocarcinoma tissue specimens. In the Cluster column, different colors indicate different clusters. In the Cancerous, Noncancerous, and Noise columns, the areas filled with blue indicate the corresponding clusters. Red and green contours indicate annotations of cancerous and noncancerous regions, respectively. Each annotation was made by a medical expert.

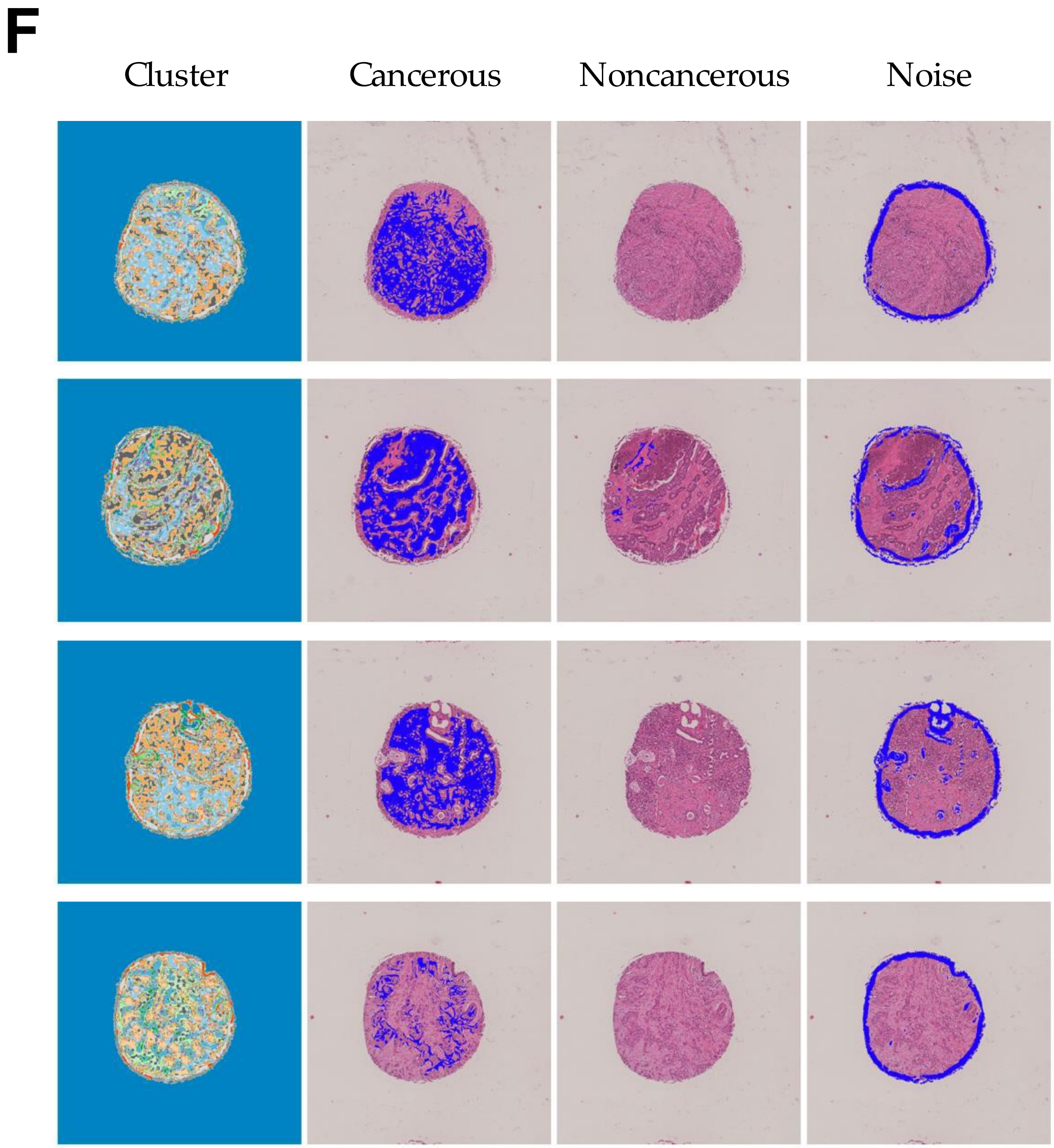

Figure S3. Clusters and annotations of human cholangiocarcinoma tissue specimens. In the Cluster column, different colors indicate different clusters. In the Cancerous, Noncancerous, and Noise columns, the areas filled with blue indicate the corresponding clusters. Red and green contours indicate annotations of cancerous and noncancerous regions, respectively. Each annotation was made by a medical expert.

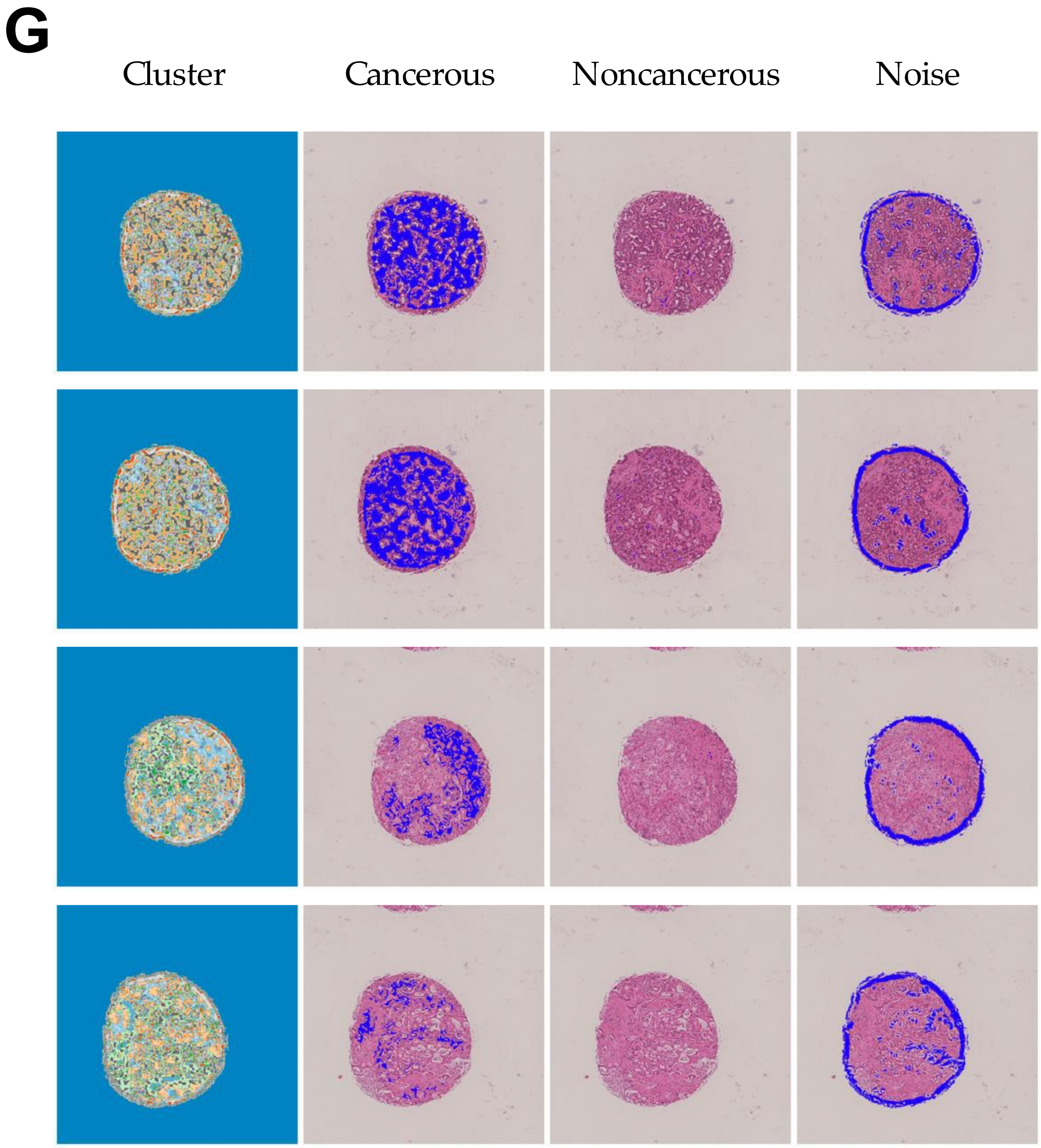

Figure S3. Clusters and annotations of human cholangiocarcinoma tissue specimens. In the Cluster column, different colors indicate different clusters. In the Cancerous, Noncancerous, and Noise columns, the areas filled with blue indicate the corresponding clusters. Red and green contours indicate annotations of cancerous and noncancerous regions, respectively. Each annotation was made by a medical expert.

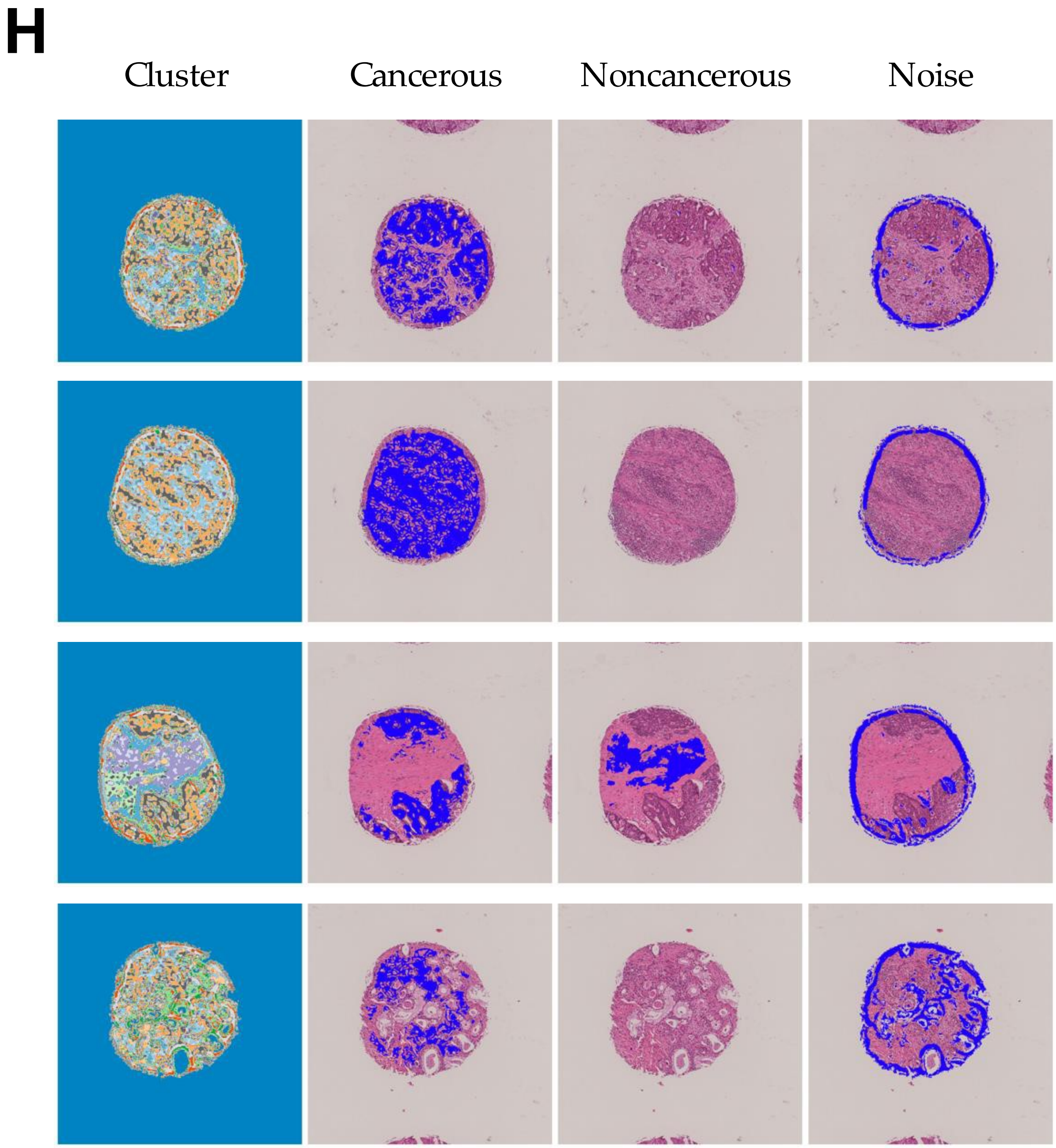

Figure S3. Clusters and annotations of human cholangiocarcinoma tissue specimens. In the Cluster column, different colors indicate different clusters. In the Cancerous, Noncancerous, and Noise columns, the areas filled with blue indicate the corresponding clusters. Red and green contours indicate annotations of cancerous and noncancerous regions, respectively. Each annotation was made by a medical expert.

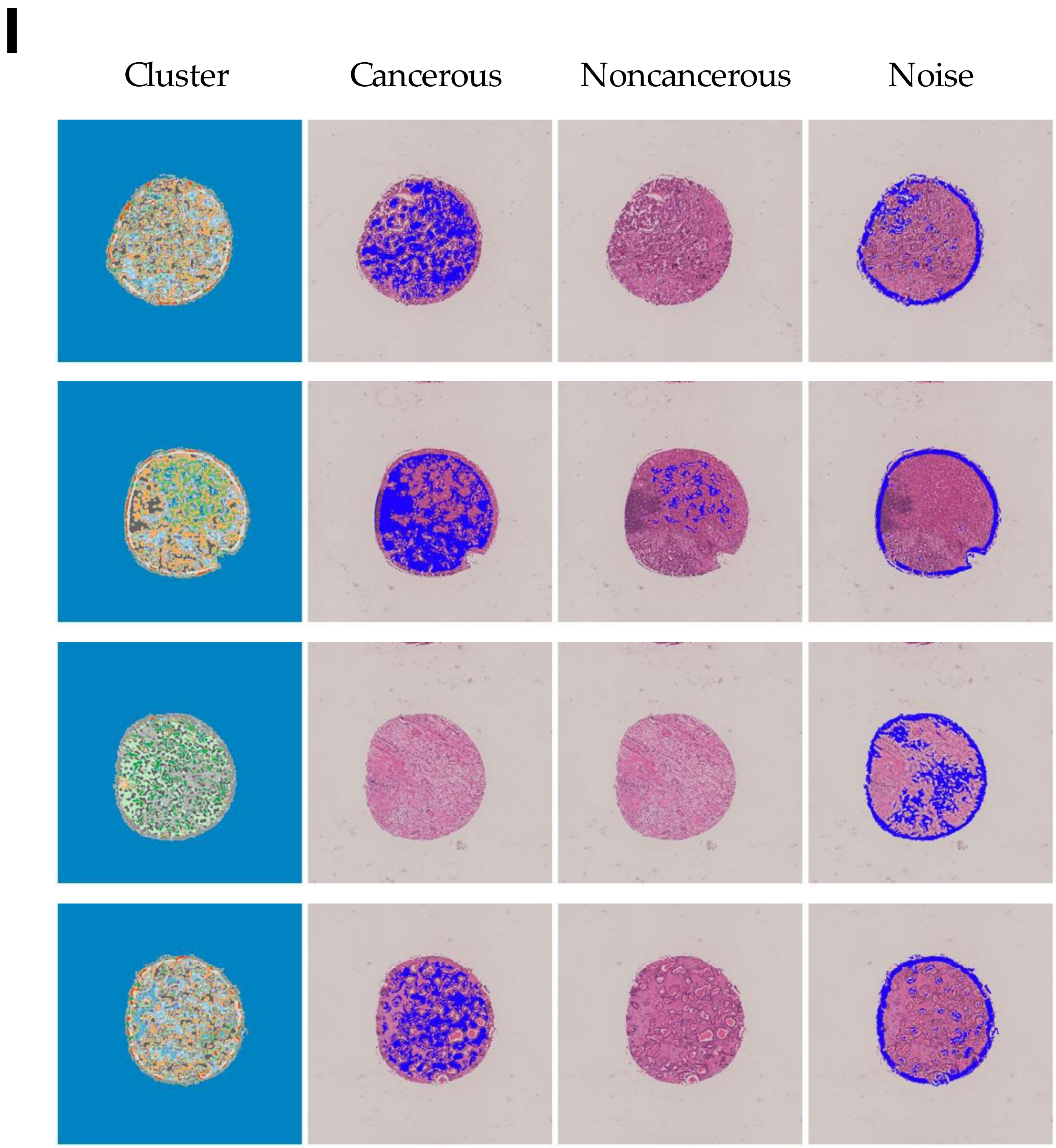

Figure S3. Clusters and annotations of human cholangiocarcinoma tissue specimens. In the Cluster column, different colors indicate different clusters. In the Cancerous, Noncancerous, and Noise columns, the areas filled with blue indicate the corresponding clusters. Red and green contours indicate annotations of cancerous and noncancerous regions, respectively. Each annotation was made by a medical expert.

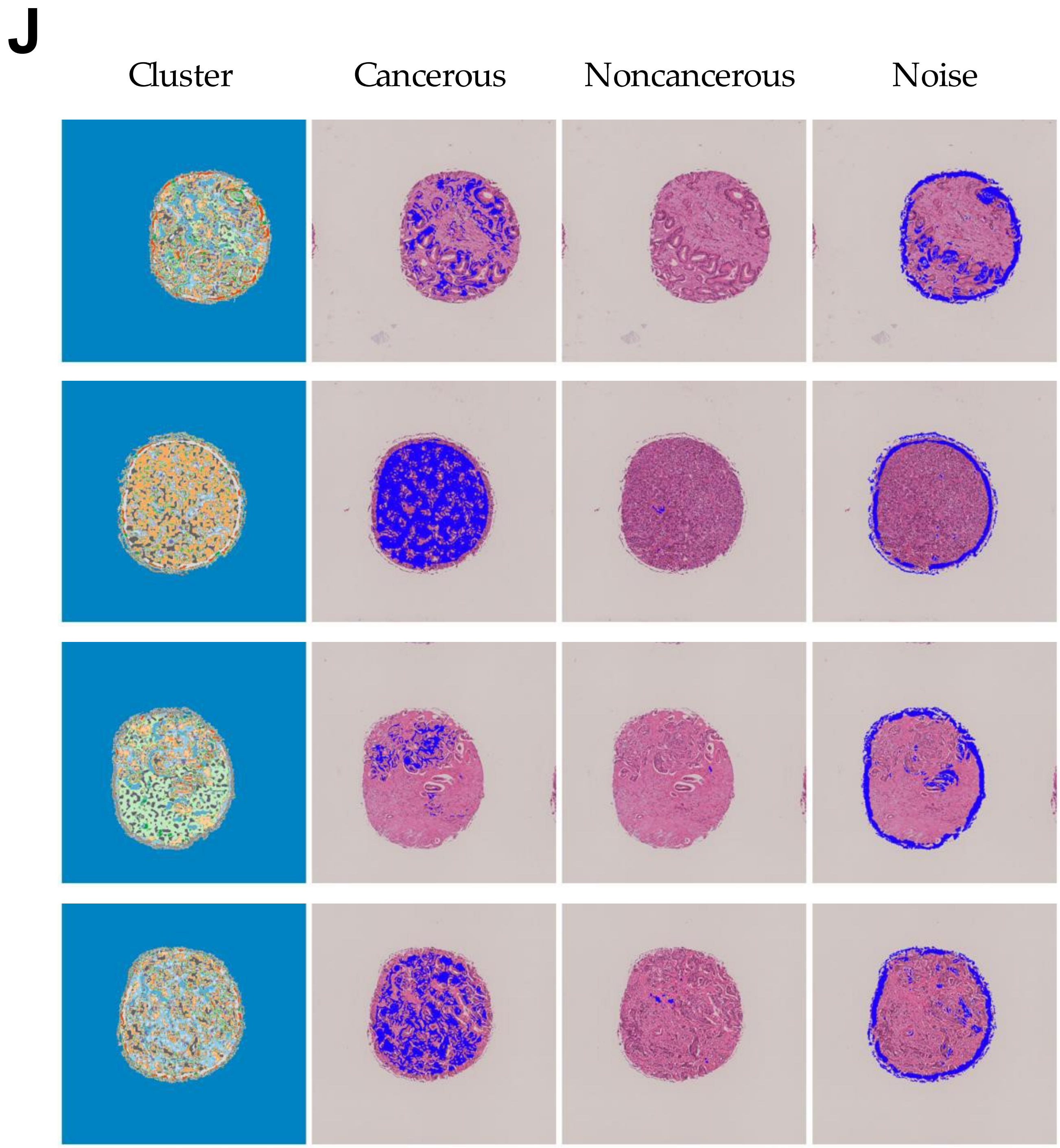

Figure S3. Clusters and annotations of human cholangiocarcinoma tissue specimens. In the Cluster column, different colors indicate different clusters. In the Cancerous, Noncancerous, and Noise columns, the areas filled with blue indicate the corresponding clusters. Red and green contours indicate annotations of cancerous and noncancerous regions, respectively. Each annotation was made by a medical expert.

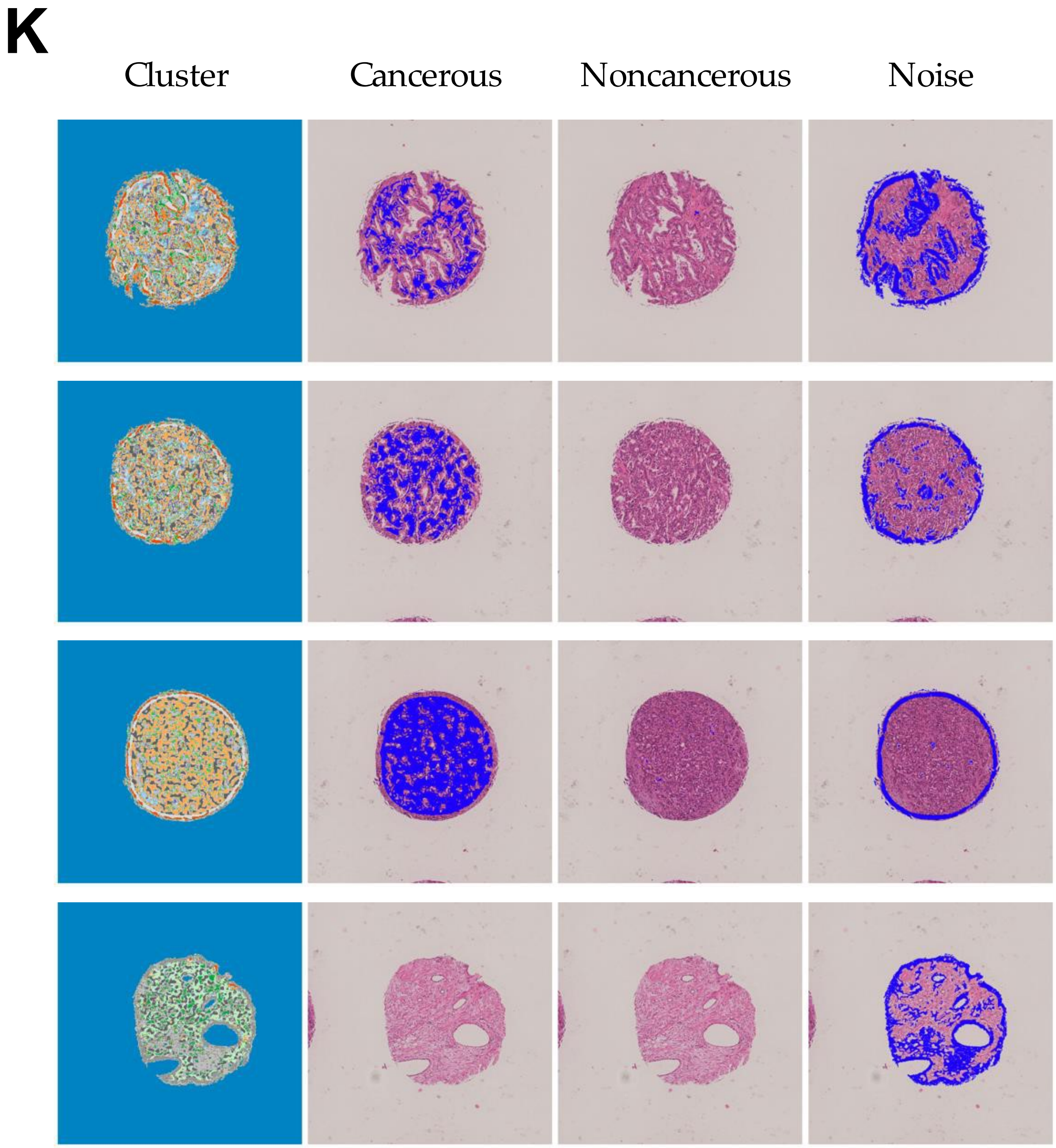

Figure S3. Clusters and annotations of human cholangiocarcinoma tissue specimens. In the Cluster column, different colors indicate different clusters. In the Cancerous, Noncancerous, and Noise columns, the areas filled with blue indicate the corresponding clusters. Red and green contours indicate annotations of cancerous and noncancerous regions, respectively. Each annotation was made by a medical expert.

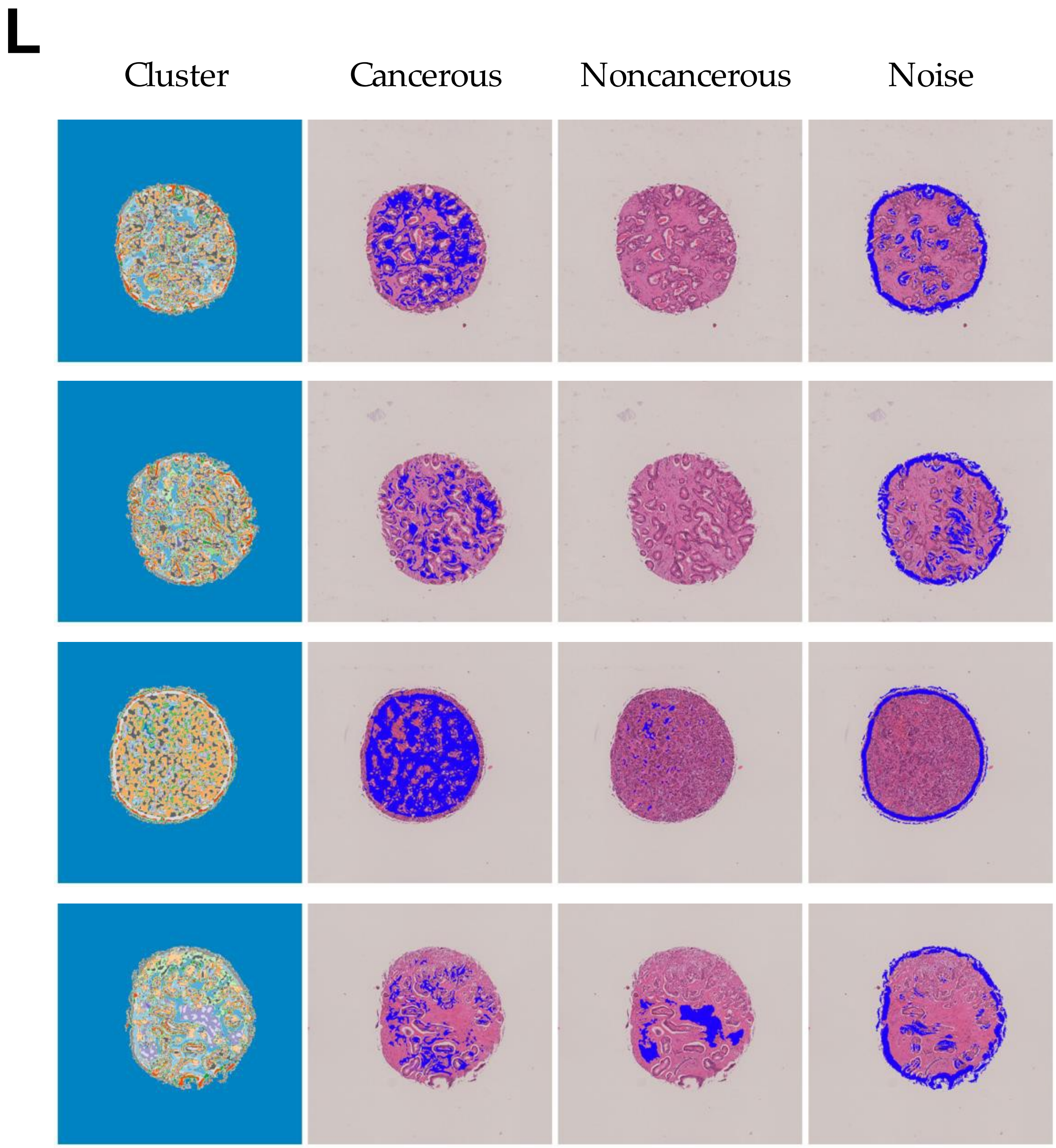

Figure S3. Clusters and annotations of human cholangiocarcinoma tissue specimens. In the Cluster column, different colors indicate different clusters. In the Cancerous, Noncancerous, and Noise columns, the areas filled with blue indicate the corresponding clusters. Red and green contours indicate annotations of cancerous and noncancerous regions, respectively. Each annotation was made by a medical expert.

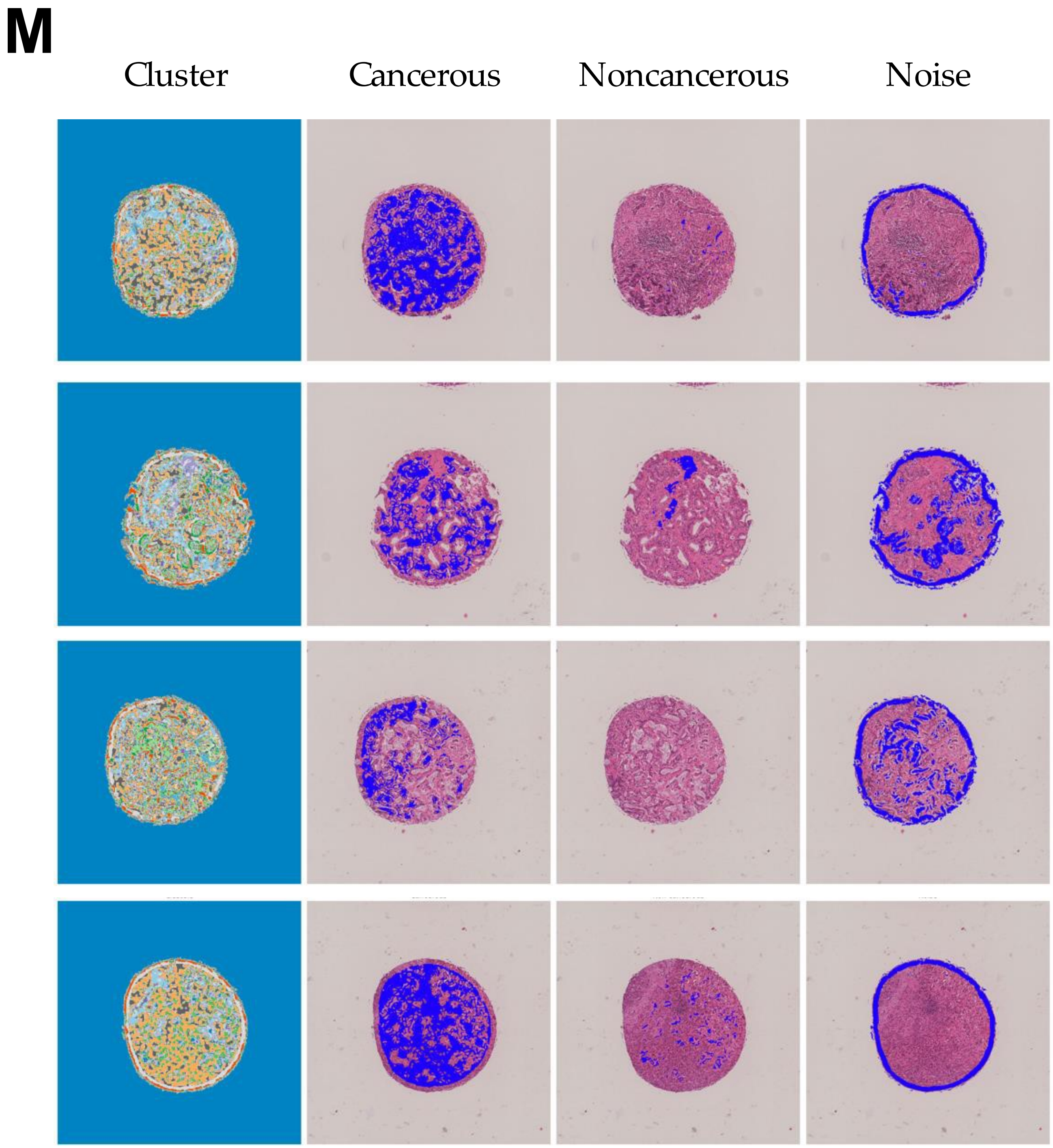

Figure S3. Clusters and annotations of human cholangiocarcinoma tissue specimens. In the Cluster column, different colors indicate different clusters. In the Cancerous, Noncancerous, and Noise columns, the areas filled with blue indicate the corresponding clusters. Red and green contours indicate annotations of cancerous and noncancerous regions, respectively. Each annotation was made by a medical expert.

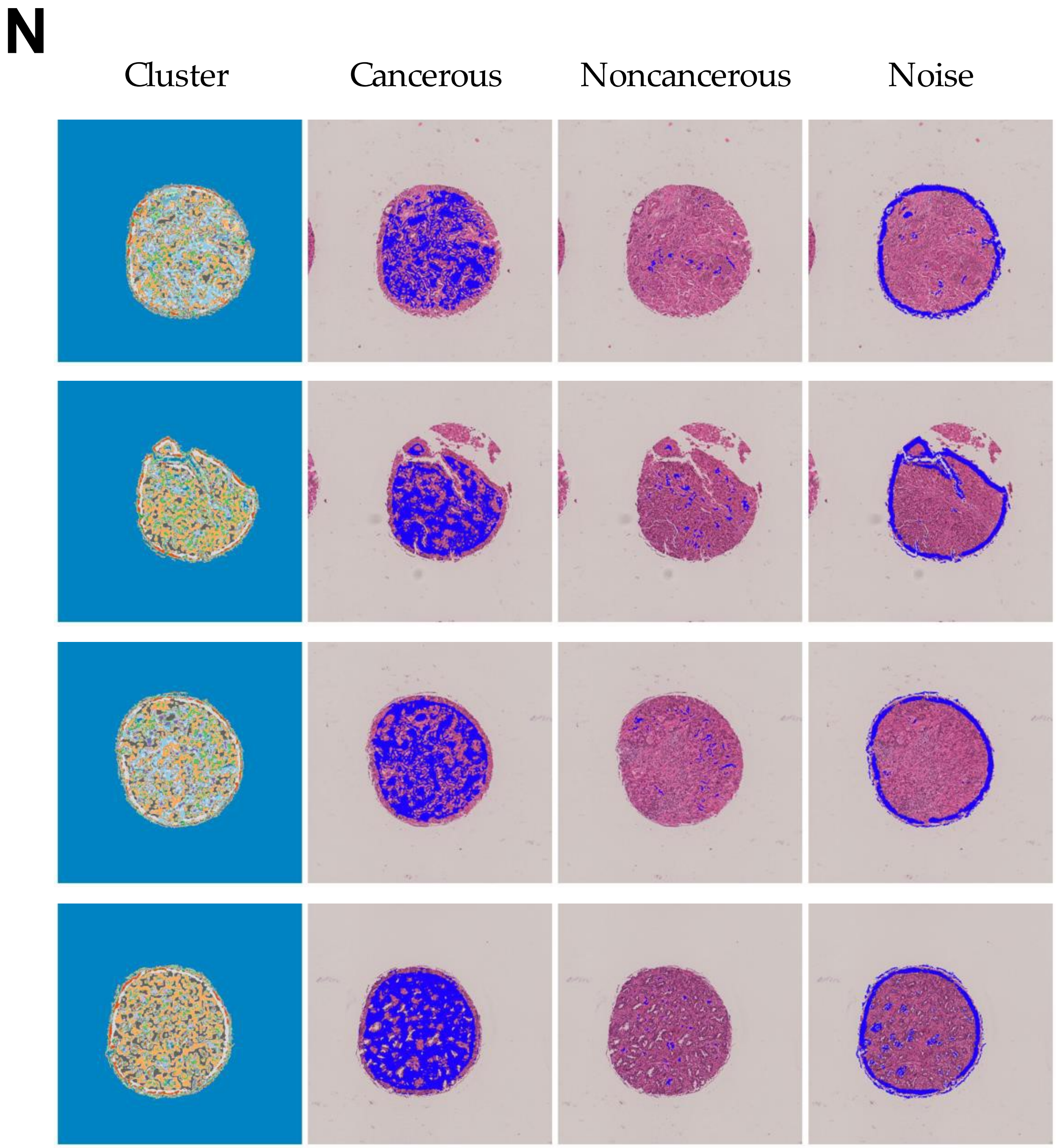

Figure S3. Clusters and annotations of human cholangiocarcinoma tissue specimens. In the Cluster column, different colors indicate different clusters. In the Cancerous, Noncancerous, and Noise columns, the areas filled with blue indicate the corresponding clusters. Red and green contours indicate annotations of cancerous and noncancerous regions, respectively. Each annotation was made by a medical expert.

O

Cluster | Cancerous | Noncancerous | Noise

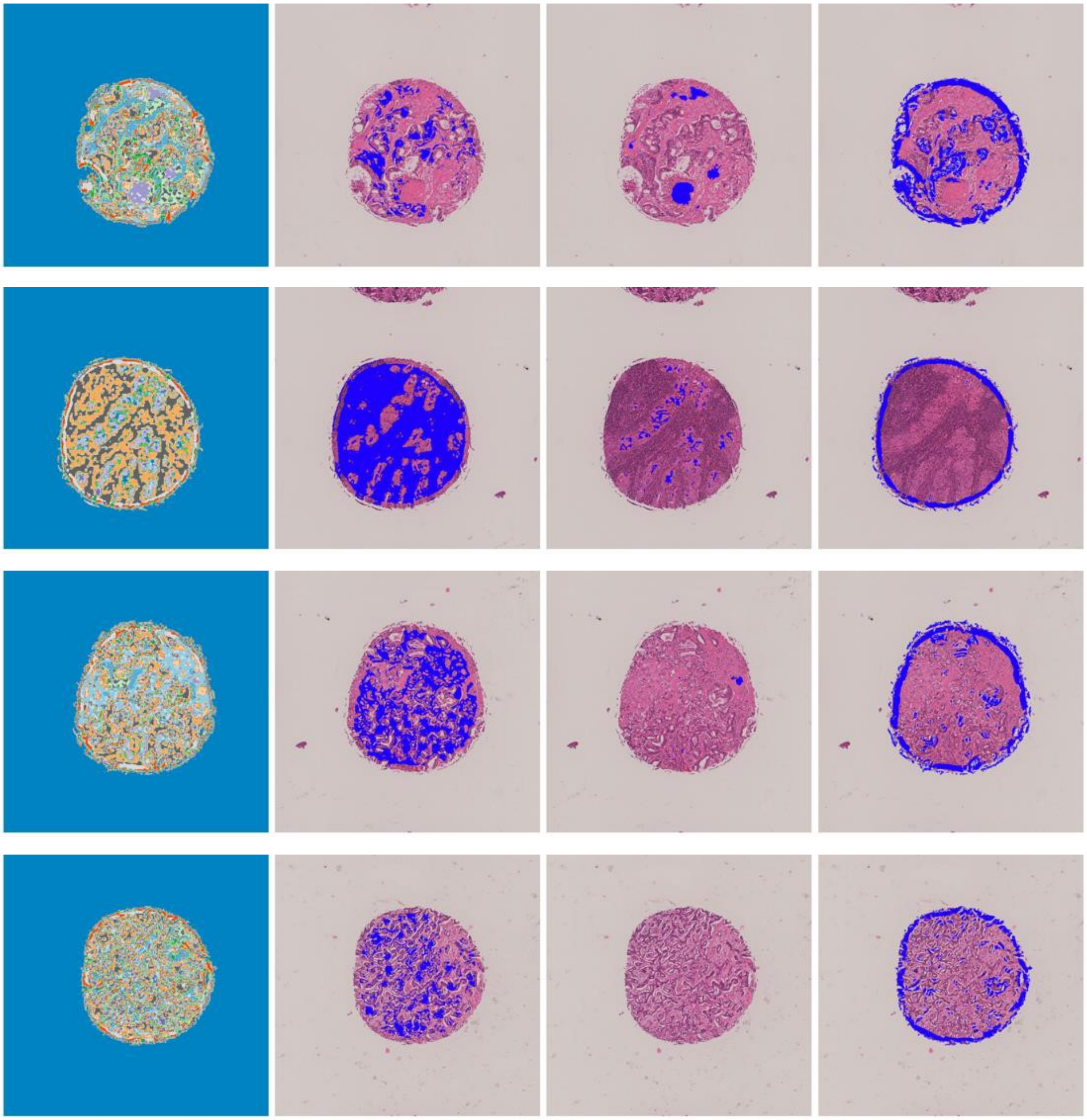

Figure S3. Clusters and annotations of human cholangiocarcinoma tissue specimens. In the Cluster column, different colors indicate different clusters. In the Cancerous, Noncancerous, and Noise columns, the areas filled with blue indicate the corresponding clusters. Red and green contours indicate annotations of cancerous and noncancerous regions, respectively. Each annotation was made by a medical expert.

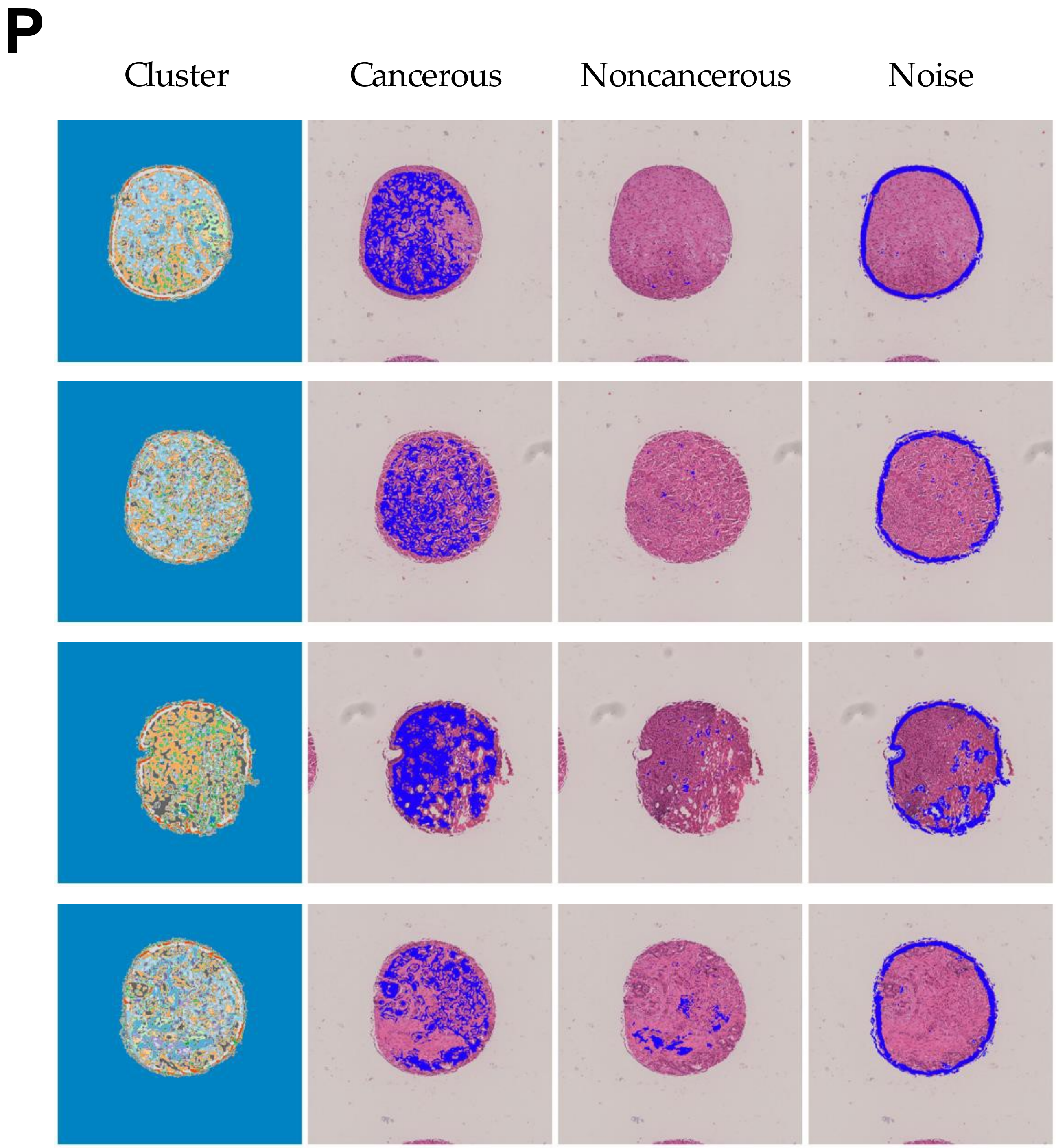

Figure S3. Clusters and annotations of human cholangiocarcinoma tissue specimens. In the Cluster column, different colors indicate different clusters. In the Cancerous, Noncancerous, and Noise columns, the areas filled with blue indicate the corresponding clusters. Red and green contours indicate annotations of cancerous and noncancerous regions, respectively. Each annotation was made by a medical expert.

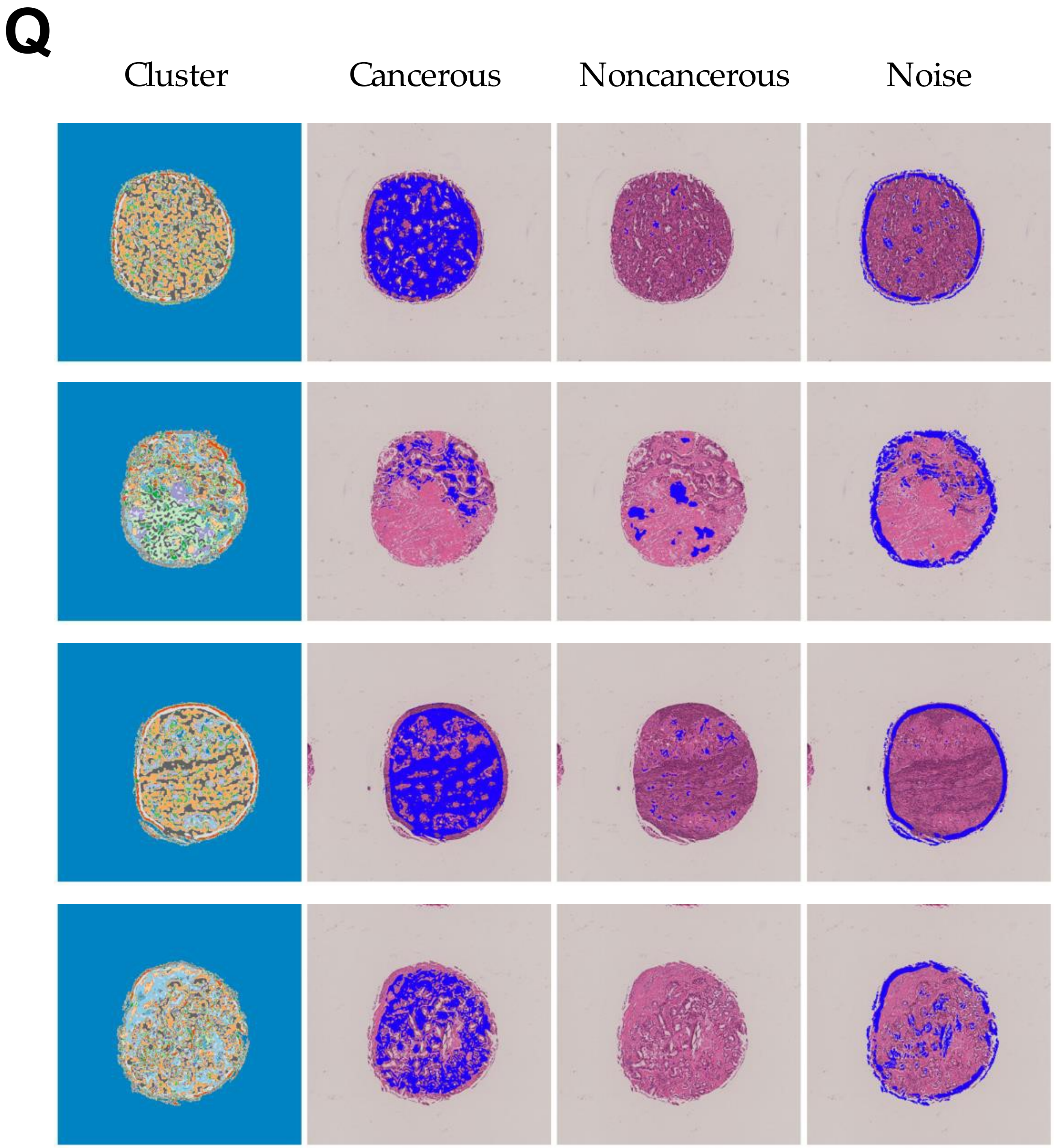

Figure S3. Clusters and annotations of human cholangiocarcinoma tissue specimens. In the Cluster column, different colors indicate different clusters. In the Cancerous, Noncancerous, and Noise columns, the areas filled with blue indicate the corresponding clusters. Red and green contours indicate annotations of cancerous and noncancerous regions, respectively. Each annotation was made by a medical expert.

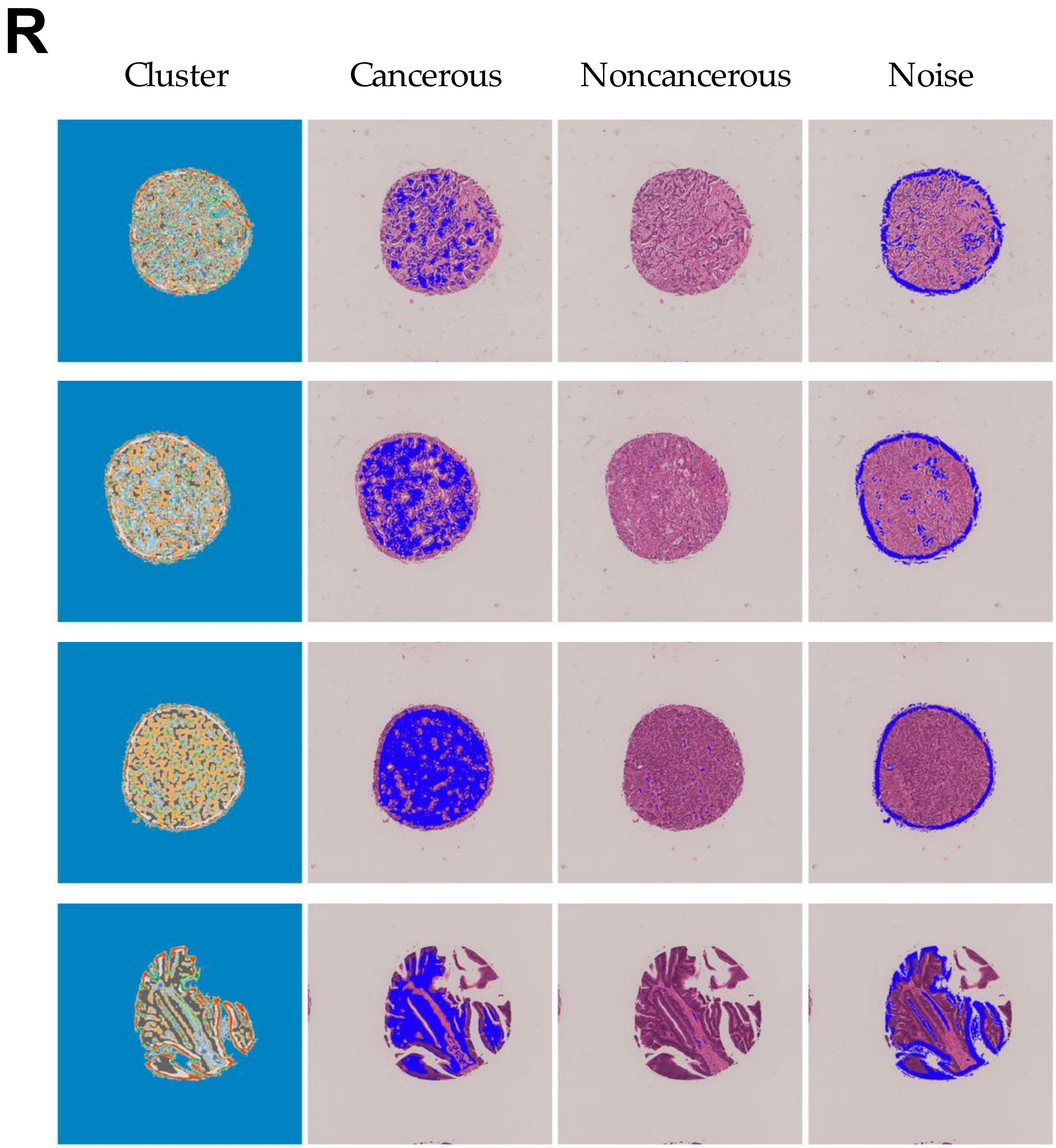

Figure S3. Clusters and annotations of human cholangiocarcinoma tissue specimens. In the Cluster column, different colors indicate different clusters. In the Cancerous, Noncancerous, and Noise columns, the areas filled with blue indicate the corresponding clusters. Red and green contours indicate annotations of cancerous and noncancerous regions, respectively. Each annotation was made by a medical expert.

S

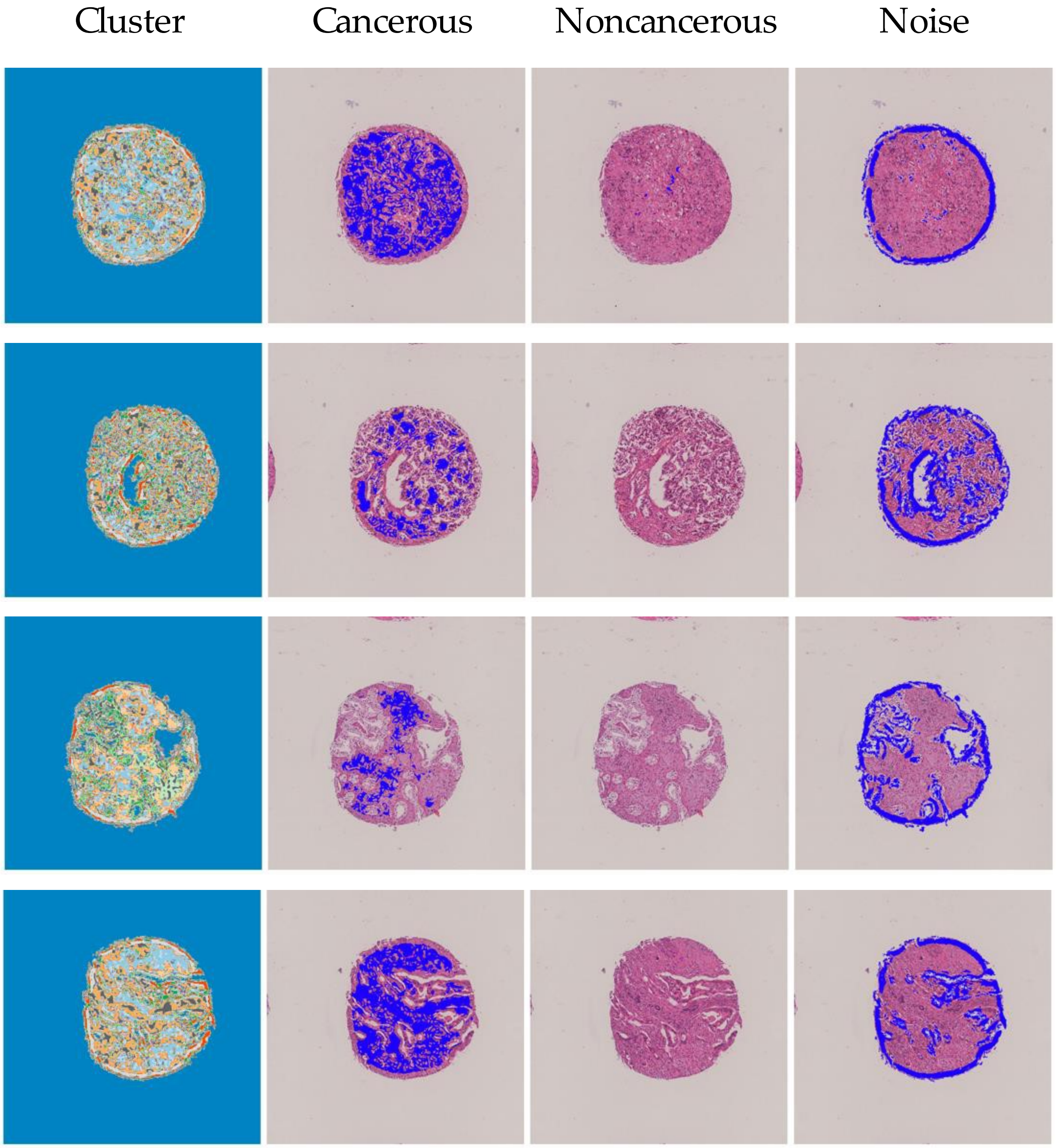

Figure S3. Clusters and annotations of human cholangiocarcinoma tissue specimens. In the Cluster column, different colors indicate different clusters. In the Cancerous, Noncancerous, and Noise columns, the areas filled with blue indicate the corresponding clusters. Red and green contours indicate annotations of cancerous and noncancerous regions, respectively. Each annotation was made by a medical expert.

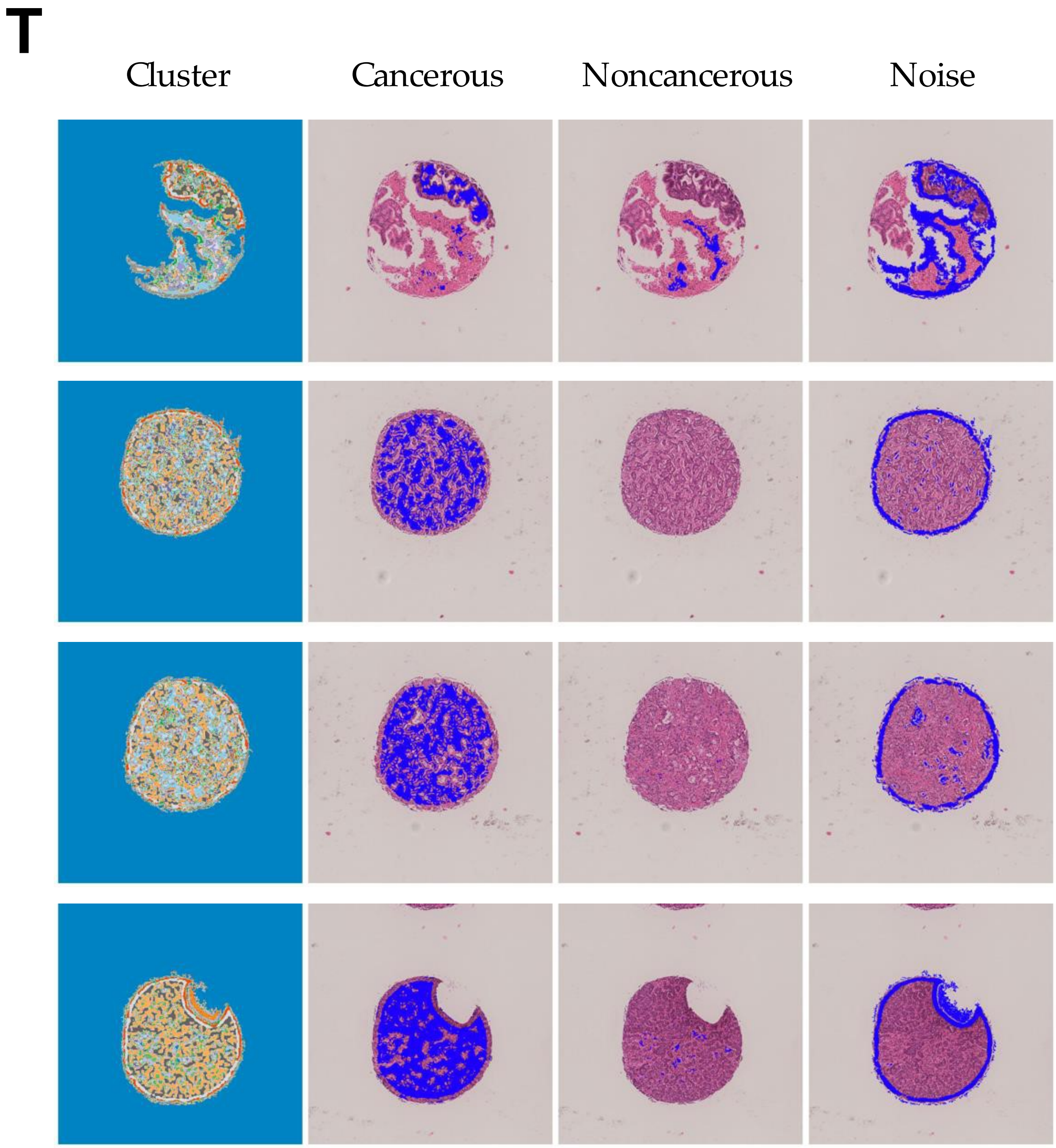

Figure S3. Clusters and annotations of human cholangiocarcinoma tissue specimens. In the Cluster column, different colors indicate different clusters. In the Cancerous, Noncancerous, and Noise columns, the areas filled with blue indicate the corresponding clusters. Red and green contours indicate annotations of cancerous and noncancerous regions, respectively. Each annotation was made by a medical expert.

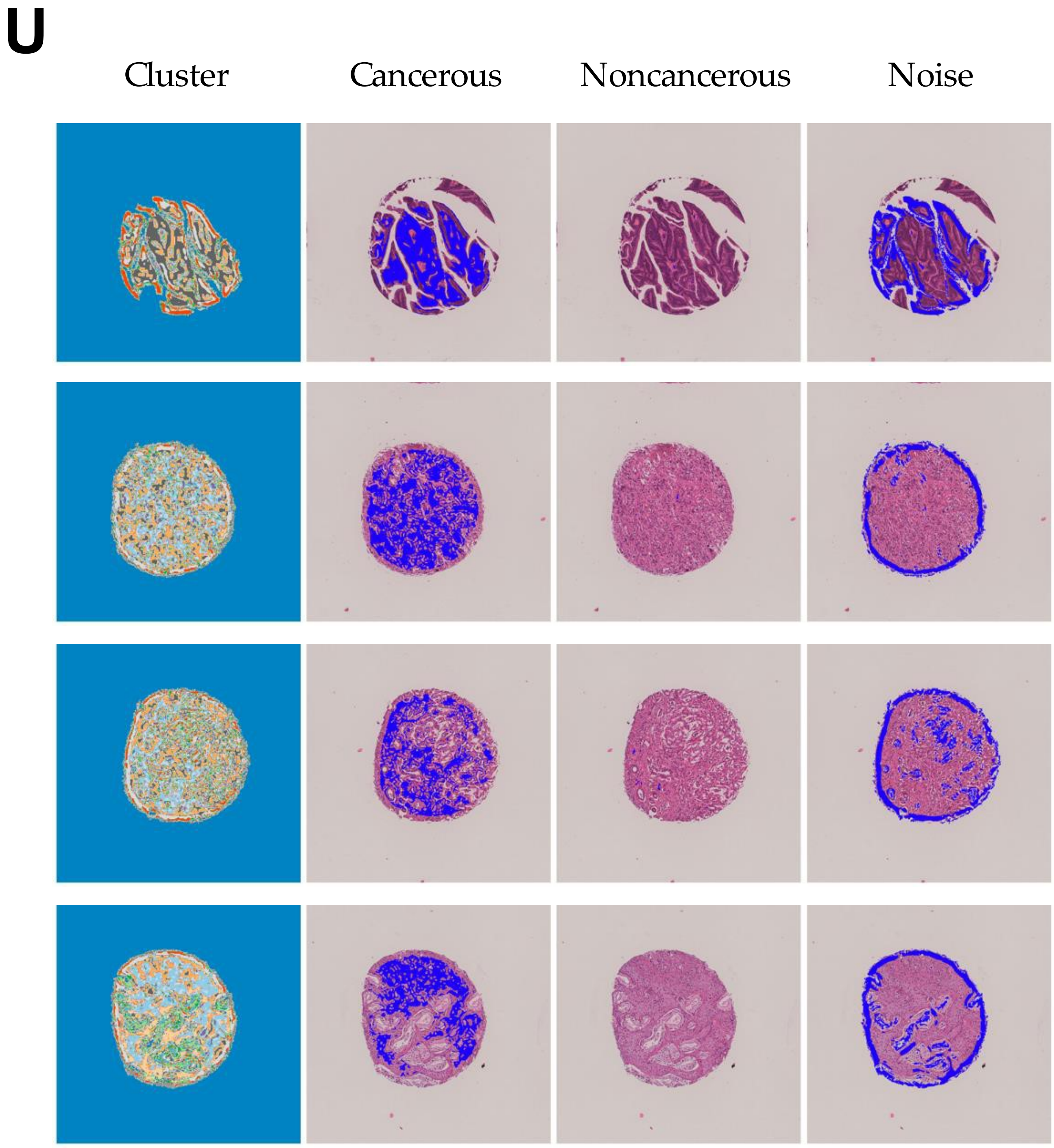

Figure S3. Clusters and annotations of human cholangiocarcinoma tissue specimens. In the Cluster column, different colors indicate different clusters. In the Cancerous, Noncancerous, and Noise columns, the areas filled with blue indicate the corresponding clusters. Red and green contours indicate annotations of cancerous and noncancerous regions, respectively. Each annotation was made by a medical expert.

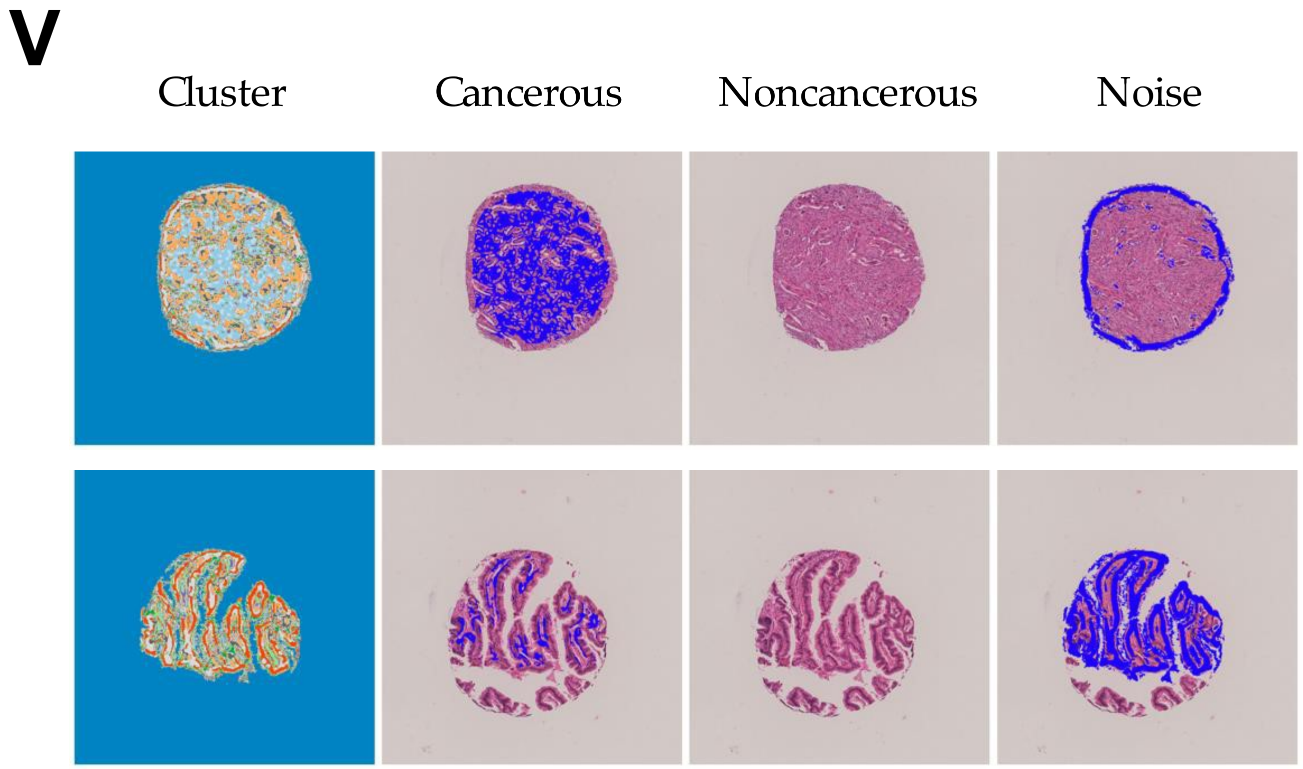

Figure S3. Clusters and annotations of human cholangiocarcinoma tissue specimens. In the Cluster column, different colors indicate different clusters. In the Cancerous, Noncancerous, and Noise columns, the areas filled with blue indicate the corresponding clusters. Red and green contours indicate annotations of cancerous and noncancerous regions, respectively. Each annotation was made by a medical expert.
